# Electrical detection of spin pumping in van der Waals ferromagnetic Cr_2_Ge_2_Te_6_ with low magnetic damping

**DOI:** 10.1038/s41467-023-39529-8

**Published:** 2023-06-28

**Authors:** Hongjun Xu, Ke Jia, Yuan Huang, Fanqi Meng, Qinghua Zhang, Yu Zhang, Chen Cheng, Guibin Lan, Jing Dong, Jinwu Wei, Jiafeng Feng, Congli He, Zhe Yuan, Mingliang Zhu, Wenqing He, Caihua Wan, Hongxiang Wei, Shouguo Wang, Qiming Shao, Lin Gu, Michael Coey, Youguo Shi, Guangyu Zhang, Xiufeng Han, Guoqiang Yu

**Affiliations:** 1 Beijing National Laboratory for Condensed Matter Physics, Institute of Physics, University of Chinese Academy of Sciences, Chinese Academy of Sciences, Beijing, 100190 China; 2 Songshan Lake Materials Laboratory, Dongguan, Guangdong 523808 China; 3 Center of Materials Science and Optoelectronics Engineering, University of Chinese Academy of Sciences, Beijing, 100049 China; 4 Advanced Research Institute of Multidisciplinary Science, Beijing Institute of Technology, Beijing, 100081 China; 5 State Key Laboratory of New Ceramics and Fine Processing, School of Materials Science and Engineering, Tsinghua University, Beijing, 100084 China; 6 Key Laboratory of Magnetism and Magnetic Materials of the Ministry of Education, School of Physical Science and Technology, Lanzhou University, Lanzhou, 730000 China; 7 Institute of Advanced Materials, Beijing Normal University, Beijing, 100875 China; 8 Department of Physics, Beijing Normal University, Beijing, 100875 China; 9 Ningbo Institute of Materials Technology and Engineering, Chinese Academy of Sciences, Ningbo, 315201 China; 10 School of Materials Science and Engineering, Anhui University, Hefei, 230601 China; 11 Department of Electronic and Computer Engineering, Hong Kong University of Science and Technology, Kowloon Hong Kong, China; 12 School of Physics and CRANN, Trinity College, Dublin, 2 Ireland

**Keywords:** Spintronics, Magnetic properties and materials

## Abstract

The discovery of magnetic order in atomically-thin van der Waals materials has strengthened the alliance between spintronics and two-dimensional materials. An important use of magnetic two-dimensional materials in spintronic devices, which has not yet been demonstrated, would be for coherent spin injection via the spin-pumping effect. Here, we report spin pumping from Cr_2_Ge_2_Te_6_ into Pt or W and detection of the spin current by inverse spin Hall effect. The magnetization dynamics of the hybrid Cr_2_Ge_2_Te_6_/Pt system are measured, and a magnetic damping constant of ~ 4–10 × 10^−4^ is obtained for thick Cr_2_Ge_2_Te_6_ flakes, a record low for ferromagnetic van der Waals materials. Moreover, a high interface spin transmission efficiency (a spin mixing conductance of 2.4 × 10^19^/m^2^) is directly extracted, which is instrumental in delivering spin-related quantities such as spin angular momentum and spin-orbit torque across an interface of the van der Waals system. The low magnetic damping that promotes efficient spin current generation together with high interfacial spin transmission efficiency suggests promising applications for integrating Cr_2_Ge_2_Te_6_ into low-temperature two-dimensional spintronic devices as the source of coherent spin or magnon current.

## Abstract (web summary)

Spin-pumping experimental technique where a DC or AC spin current is generated, and typically transferred to a heavy metal layer where it can be detected via electrical measurements. While well established in conventional materials, coherent spin-pumping in van der Waals magnetic materials is challenging due to the low damping and high-quality interface requirements. Here, Xu et al demonstrate coherent spin pumping in the van der Waals magnet Cr2Ge2Te6.

## Introduction

Magnetic two-dimensional (2D) van der Waals (vdW) systems have recently become a vibrant research field because of their exotic properties and central role in building 2D spintronic devices^1–5^. The spin injection is one of the most important aspects of spintronics, which lays the foundation for studying spin transport and relaxation in the low-dimensional systems in relation to other quantities like charge, valleys, lattice or band topology^6–9^. Making use of the magnetic 2D materials, people have realized spin injection into the nonmagnetic vdW materials through spin-polarized electron tunnelling^10,11^ and thermally-driven spin injection^10,12^. Beyond these methods, spin pumping driven by ferromagnetic resonance (FMR) is another well-established scheme^13^, and it has been widely used for spin injection from conventional magnetic materials into a great number of different systems: metals^14^, semiconductors^15^, topological insulators^16,17^, superconductors^18^ and quantum materials^19^. The main advantage of using spin-pumping lies in the coherent generation of both pure d.c. and a.c. spin currents with high efficiency, free from impedance mismatch problems^20,21^. Therefore, considering the vast number of 2D materials, spin pumping based on vdW magnetic materials could significantly advance their applications in spintronic devices. This will not only benefit studies of the various spin-related phenomena in nonmagnetic vdW materials but also, in turn, the spin dynamic properties of the magnetic vdW systems. However, the use of magnetic vdW materials for coherent spin-pumping and its electrical detection has not yet been demonstrated. Two factors might hamper the development of this technique: suitable vdW magnets with relatively low magnetic damping and high-quality interfaces for efficient transfer of spin angular momentum to adjacent materials. Closely related questions are whether the spin dynamic behaviour of the vdW magnet at the 2D limit (with strong spin fluctuations) deviates from that of an ultra-thin conventional magnet and how efficient the spin current transfer can be across the vdW gaps.

**Fig. 1:**
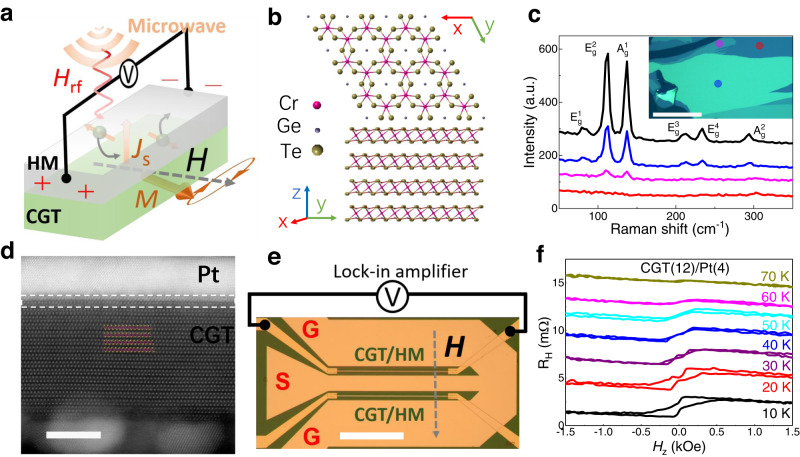
Schematic diagram of the spin-pumping device based on vdW Cr_2_Ge_2_Te_6_ (CGT). **a** Schematic illustration of the spin-pumping experimental set-up, where *H*_rf_ and *J*_s_ stand for the oscillating magnetic field and d.c. spin current, respectively. **b** Atomic structure of CGT from top and side views, where a central Cr atom (magenta sphere) bonded to six ligand atoms (Te) consisting of a honeycomb network of edge-shared CrTe_6_ octahedra elongated by Ge dimers. **c** Raman spectra for various CGT/Pt thin films, which are detected from the spots shown in the inset (the red, magenta, blue and black colors correspond to the stacks with monolayer, bilayer, four-layer, and bulk CGT, respectively). **d** High-angle annular dark-field STEM image of the cross-section of a CGT/Pt bilayer, its interface region is marked by a white dashed rectangle with Au particles beneath the CGT. **e** The spin-pumping device layout, where the RF signal is introduced via the left side of Ground-Source-Ground (GSG) pads (which are isolated from CGT/HM stacks by a SiO_2_ layer), an oscillating RF magnetic field was generated perpendicular to the CGT stack. **f** Typical Hall resistance of a CGT/Pt bilayer, where the vanishing anomalous Hall resistance between 60 K and 70 K signifies a Curie temperature of around ~65 K. The scale bars are 200 µm, 5 nm, and 200 µm in (**c**), (**d**), and (**e**), respectively.

Here, we report efficient spin pumping from a layered ferromagnetic insulator, Cr_2_Ge_2_Te_6_ (CGT), into a heavy metal (HM), as shown in Fig. 1a. A state-of-the-art low magnetic damping constant was obtained in the CGT/Pt bilayer from the frequency-dependent linewidth of the spin-pumping signals. This property suggests the CGT crystal can be a promising host material for transporting spin or magnon current (which can be excited incoherently or coherently) over long distances. The interface properties of CGT/Pt were directly investigated from the dependence of magnetic damping on CGT thickness, and the spin mixing conductance was deduced. The magnetic damping constants and interface spin mixing conductance of the CGT-based system at low temperatures are comparable to those of the benchmark yttrium iron garnet (YIG) spin-pumping system with ultra-low magnetic damping at room temperature^22^. Moreover, in contrast to the fact that the spin-pumping efficiency of the YIG thin film-based system usually worsens when cooling below room temperature, the spin-pumping efficiency in CGT/Pt exhibits only a mild temperature dependence below 50 K. The CGT-based system is more appealing for coherent temperature-independent spin pumping. Our work reveals that CGT can efficiently generate dynamic spin currents and deliver them across the interface, which makes CGT an ideal 2D platform for studying spin dynamics in a low-dimensional hybrid system at low temperatures. For instance, using CGT as a coherent spin source and integrating it with a nonmagnetic transition metal dichalcogenide (TMD), one might seamlessly study the interplay between spin and other degrees of freedom including the valleys, symmetry, and twist angle of the heterostructures^23,24^. Although the working temperature of these heterostructures is currently limited by the relatively low Curie temperature of pristine CGT, our work highlights the potential of using CGT and other 2D magnets (including ferromagnets and antiferromagnets) as the coherent spin/magnon source in full-vdW spintronic devices and exploring emerging phenomena and device functions at the designed atomic interfaces and hybrid structures etc. Further understanding of the underlying mechanisms for low magnetic damping in CGT and looking deep into the spin relaxation process across the interface helps to resolve the challenges of developing more compact and versatile spintronics devices based on vdW materials in the future.

## Results

### Sample characterization and device fabrication

As one of the first-reported intrinsic 2D ferromagnets (FM), CGT has attracted tremendous attention^1,25–28^. Below *T*_c_ (~67 K for bulk material), the neighboring Cr atoms are ferromagnetically coupled to each other through Cr-Te-Cr bonds with a bond angle of ~90^o^ (Fig. 1b). Being a nearly ideal 2D Heisenberg system, the magnetocrystalline anisotropy energy required for long-range ferromagnetic order is generated by a covalent bond between ligand Te-p and Cr-e_g_ orbitals^29^. The magnetic properties of the bulk CGT used in this work are similar to those reported in the literature (Supplementary Fig. S1)^30^.

Thanks to the Au-assisted exfoliation method^31^, we managed to exfoliate bulk CGT into sub-millimeter size flakes of various thicknesses (inset of Fig. 1c and Methods). The final step of the exfoliation process was carried out in a high-vacuum chamber to avoid oxidizing the CGT surface^32^. The HM (Pt or W) thin films were subsequently grown onto the fresh surface of CGT by low-power sputtering (see Methods). The HM layer serves as the spin current detector via the inverse spin Hall effect (ISHE). Although we tried avoid it, some damage to the CGT surface during HM deposition was inevitable. For a single CGT monolayer covered by Pt (marked by a red dot and corresponding to the red curve in Fig. 1c), no characteristic 2D Raman spectrum is detectable, but for relatively thicker CGT flakes (two or more layers), the characteristic E_g_^2^ and A_g_^2^ Raman modes show up without any sign of oxidation (Methods)^33^. Figure 1d shows the cross-section of the CGT/Pt stack, acquired by aberration-corrected scanning transmission electron microscopy (STEM). Only the CGT/Pt interface layer is imperfect (see the white dashed rectangle region in Fig. 1d). The layered CGT beneath stays intact, consistent with the Raman spectra.

Figure 1e shows the device fabricated for the spin pumping measurement (see the other device geometry in Supplementary Fig. S6a). CGT/HM bilayers with a typical length of 300 µm were used (see the two CGT/HM stacks in Fig. 1e), which are capable of observing spin-pumping voltages (*V*_sp_) with a high signal-noise ratio. The Hall bar devices were fabricated at the same time as the spin-pumping devices on the sub-mm flakes. The magnetic properties of the insulating CGT flakes are first probed by the anomalous Hall effect (AHE) in the CGT/Pt and CGT/W Hall-bar devices, as shown for Pt in Fig. 1f. For the CGT(12)/Pt(4), clear AHE signals are found below 70 K, which indicates that the *T*_c_ of 12 nm CGT is ~ 65 K (numbers in brackets throughout this paper are in nanometers). The *T*_c_ is consistent with the magnetic properties of bulk CGT with out-of-plane uniaxial magnetic anisotropy and also agrees with the previous reports of CGT/Pt^34,35^. Similarly, clear AHE signals are also observed in CGT/W bilayers (Supplementary Fig. S4). The observation of AHE proves the transfer of spin current through the CGT/HM interface, which prompts us to perform spin-pumping measurements in CGT/HM devices.

### Electrical detection of spin-pumping in CGT/Pt and CGT/W

**Fig. 2:**
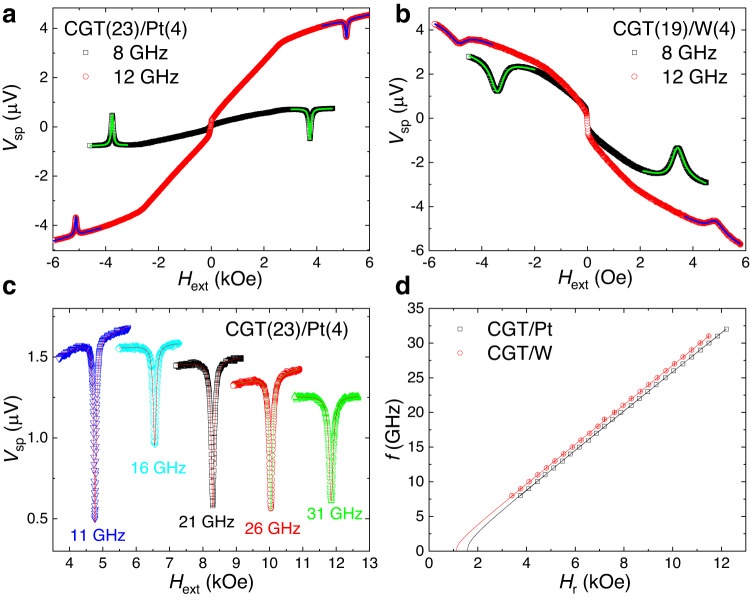
Representative spin-pumping results from CGT/Pt and CGT/W. **a** Typical signals from the CGT(23)/Pt(4) stack at 30 K under RF signal frequencies of 8 GHz and 12 GHz, the spin-pumping voltages (*V*_sp_) with linewidth (Δ*H*) and resonance field (*H*_r_) are extracted by fitting a Lorentzian function with a linear background. **b** Spin-pumping voltages from CGT(19)/W(4) stack where the *V*_sp_ is inverted as compared with CGT/Pt. **c** A series of *V*_sp_ from CGT/Pt device under a wide range of RF signal frequencies (8 − 40 GHz). **d** The resonance field as the function of RF frequency is nicely fitted with Kittel’s equation for CGT/Pt and CGT/W individually at 30 K.

Figure 2a shows typical results for *V*_sp_ as the function of the external magnetic field for CGT/Pt at 30 K. It is noted that the probing direction, spin current, and magnetization orientation are mutually orthogonal in order to maximize the spin-pumping voltages (while their directions are different in the second device geometry, see Supplementary Fig. S6). In this case, *V*_sp_ can be calculated as^36,37^.

1 $${V}_{{SP}}={e\theta }_{{SH}}{l}_{{sf}}{LR}\tanh \left(\frac{{t}_{{NM}}}{2{l}_{{sf}}}\right)\,{{f Pg}_{{eff}}^{\uparrow \downarrow}} {\sin}^{2} \Theta $$

Here, *e* is the electron charge, ${\theta }_{{SH}}$ is the spin Hall angle, ${l}_{{sf}}$ is the spin diffusion length, *L* is the probe length, *R* is the resistance of the HM, *f* is the microwave frequency, *P* is the ellipticity correction factor, and ${g}_{{eff}}^{\uparrow \downarrow }$ is the effective interface spin mixing conductance. $\Theta$ presents the precession cone angle, $\Theta={h}_{{rf}}/\triangle H\ll 1$, where ${h}_{{rf}}$ and $\triangle H$ are the microwave magnetic field and FMR linewidth. Note that linewidth scales linearly with the effective magnetic damping (see below Eq. 2). It is clear from this formula that under fixed RF power and parameters of spin detector, the *V*_sp_ (spin-pumping efficiency) is proportional to the effective spin mixing conductance and is approximately inversely proportional to the square of the magnetic damping.

Characteristic Lorentzian peaks emerge when scanning the in-plane magnetic field *H* for different microwave frequencies. The resonance peaks can be nicely fitted by a Lorentzian function with symmetric and antisymmetric components. The symmetric component originates from the spin-pumping effect^14,36^. The antisymmetric component, which usually originates from the spin rectification effect^38^, is more than an order of magnitude smaller because of the insulating property of CGT and the low level of anisotropic magnetoresistance in CGT/Pt (Supplementary Fig. S4c, d). Therefore, the spin rectification effect can be safely ignored in our devices, which is an advantage of using an insulating FM rather than a conducting FM to obtain cleaner spin-pumping signals. The off-resonance background signal probably results from the spin Seebeck effect due to heating of the system by microwave irradiation. A pair of peaks with opposite signs of *V*_sp_ are observed for different field directions, which is the direct evidence of spin pumping^39^. The two resonance peaks have almost the same height (differences are less than 5%), which excludes the field-independence heating effect reported in spin-pumping measurements^15,40^. The sign of *V*_sp_ for CGT/W stacks is always opposite to that CGT/Pt, (Fig. 2b) under the same experimental condition. This is inferred directly from Eq. 1 because of the opposite spin Hall angles of Pt and W, which is further evidence that pure spin currents are pumped out from the CGT and injected into the adjacent Pt and W layers^39^. The peaks are wider for CGT(19)/W(4) than for CGT(23)/Pt(4), which is mainly due to the larger spin Hall angle in W and perhaps different interface properties.

Figure 2c shows representative examples of *V*_sp_ at different microwave frequencies. It is noted that a broadband (2-40 GHz) radio frequency (RF) signal can be efficiently introduced into the CGT stacks in our set-up, and *V*_sp_ is linearly proportional to the RF power when it is not too high (10-20 dBm). The different peak heights are mainly due to the frequency-dependent microwave losses and ellipticity correction factors. Plotting frequency as a function of the resonant field *H*_r_ acquired from Lorentzian fitting (Fig. 2d), one can extract the effective demagnetization field, $4\pi$*M*_eff_, of the CGT and the gyromagnetic ratio γ by the fitting of the Kittel equation:

$f=({{{{{\rm{\gamma }}}}}}/2\pi )\sqrt{{H}_{{{{{{\rm{r}}}}}}}({H}_{{{{{{\rm{r}}}}}}}+4\pi {M}_{{{{{{\rm{eff}}}}}}})}$ with ${{{{{\rm{\gamma }}}}}}={{{{{\rm{g}}}}}}{\mu }_{B}/\hslash$, $4\pi {M}_{{{{{{\rm{eff}}}}}}}=4\pi {M}_{s}-2{K}_{{{{{{\rm{u}}}}}}}/{M}_{s}$. *M*_s_ and *K*_u_ stand for saturation magnetization and out-of-plane uniaxial magnetocrystalline anisotropy constant, respectively^41^. A negative *M*_eff_ and a positive intercept on the *H*_r_ axis in the *f*-*H*_r_ plot indicate the perpendicular magnetic anisotropy (PMA) of the CGT crystals. The larger value of 4π*M*_eff_ ~ −1560 G for CGT(23)/Pt(4) than that of CGT(19)/W(4) (~ −1110 G) might originate from their different interface properties. Note that for the devices with thicker CGT, multi-peak resonance was often observed (see Supplementary Note 5) which was thus fitted by the multi-Lorentzian function. It might be related to the multi-domain modes, standing spin-wave modes, or inhomogeneity of devices, etc^40,42,43^.

### Temperature and thickness dependence of spin pumping

**Fig. 3:**
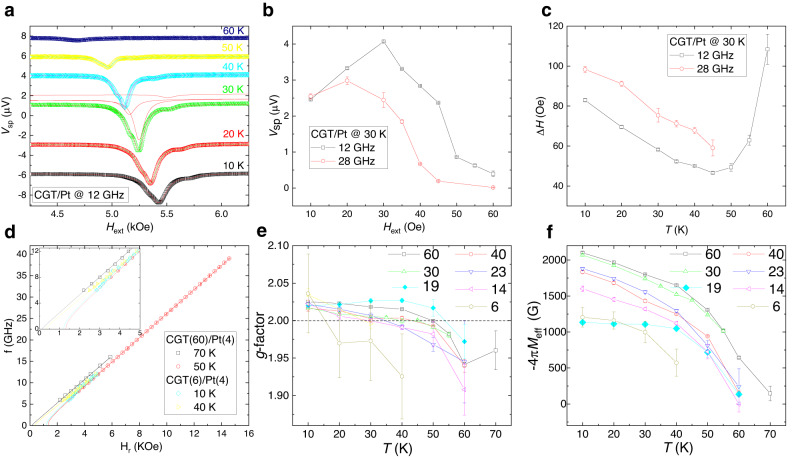
Temperature dependence of spin-pumping signals. **a** Temperature dependence of *V*_sp_ from CGT(30)/Pt(4) under 12 GHz RF excitation, where the results are fitted by three symmetric Lorentzian peaks (an example is shown for *V*_*sp*_ at 30 K). **b** The extracted *V*_sp_ of the main peak observed at 12 GHz and 28 GHz (see data on Supplementary Fig. S9) show a similar trend of temperature dependence. **c** Temperature dependence of linewidth of *V*_sp_ of the main peak at 12 GHz and 28 GHz. **d** *f-*H plots for CGT/Pt stacks with the thickest and thinnest CGT in this work. Kittel’s equation was used to fit the data points (extending to intercept with *x*-axis). The resulting *g*-factor and effective *M*_s_ of CGT are shown in (**e**) and (**f**) for CGT with different thicknesses at different temperatures. Note that the dashed black line in (**e**) marks the *g*-factor of free electron, and data for CGT(19)/W(4) are plotted as cyan curves in (**e**) and (**f**), and the solid lines in (**b**), (**c**), (**e**) and (**f**) are to guide the eye. Error bars throughout this paper correspond to standard errors.

Figure 3a shows the temperature dependence of the resonance peaks for CGT(30)/Pt(4). Clear spin-pumping signals emerge when the temperature is lower than *T*_c_. In fact, the resonance is still detectable when the temperature is slightly above the *T*_c_ (see the example of *V*_sp_ at 70 K in Supplementary Fig. S8). As the temperature decreases from slightly above *T*_c_ to well below it, *V*_sp_ changes non-monotonically, as shown in Fig. 3b for the main peak. When the temperature falls below 60 K, *V*_sp_ increases evidently, and its magnitude usually peaks at ~ 30 K and then slightly decreases with further decrease in temperature. The increasing spin-pumping signals with lowing temperature is mainly related to the development of magnetization. The decrease of *V*_sp_ in the lowest temperature regime is because of larger magnetic damping (in other words Δ*H*, see Fig. 3c), which is expected from Eq. 1. This temperature dependence of *V*_sp_ (and the linewidth) was also observed at other frequencies (see the data in Supplementary Fig. S9) with a similar trend, which is distinct from abruptly quench of *V*_sp_ with decreasing temperature in the YIG/Pt bilayer system, as we discuss later.

By fitting *V*_sp_ at different temperatures for various thicknesses of CGT, we can calculate the values of $\gamma$, *M*_eff_, and *K*_u_ for thin CGT flakes, which would be difficult to access from conventional FMR spectra because of the small volumes. Figure 3d shows the *f*-*H*_r_ plot for CGT/Pt stacks with the thickest and thinnest CGT flakes measured in this work. The smaller intercepts on the *H*_r_-axis of the plot imply a lower *K*_u_ and PMA in the thinner CGT flakes. The different slopes of the asymptotes of the *f*-*H*_r_ plots at the large resonant fields reflect the change of gyromagnetic ratio and hence *g*-factor with temperature, as shown in Fig. 3e, which characterizes the contribution of orbital angular momentum to the magnetization. It is found in CGT(60)/Pt(4) that *g* = 2 at ~50 K, and it keeps increasing as the temperature decreases from 60 K (Fig. 3e). The same trend of temperature-dependent *g*-factor was reported in bulk CGT observed by conventional FMR^42^, which confirms the fidelity of our measurement. The deviation from *g* = 2 implies the presence of orbital angular momentum in bulk CGT. It may be due to a nonspherical charge distribution in the *d* shells preventing the complete quench of the orbital moment or it may be rooted in the spin-orbit interaction of CGT^42,44^. A similar trend appears for the thinner CGT devices with relatively larger decreases of *g*-factor when the temperature is close to *T*_c_. Because the *T*_c_ of a 12 nm CGT flake is almost the same as that of bulk CGT (as shown in Fig. 1f), and the exact *M*_s_ of the CGT flakes are not detectable at this stage, it is reasonable to use the value of bulk CGT and the extracted *M*_eff_ (Fig. 3f) to calculate the anisotropy energy of these various CGT flakes. *K*_u_ is in the range (1.5–3.9) × 10^5^ erg/cm^3^ from 60 K to 10 K, which agrees with the values of bulk CGT (see Ref. ^42^. and Supplementary Fig. S1b). The larger PMA (larger magnitude of *M*_eff_) in thicker CGT at lower temperatures implies the bulk origin of PMA in CGT. However, the difference between CGT(23)/Pt(4) and CGT(19)/W(4) (with *K*_u_ ~ 3.1 × 10^5^ and 2.7 × 10^5^ erg/cm^3^ respectively at 10 K) indicates a contribution of the interfaces to the PMA.

### Magnetic damping constant and spin mixing conductance of CGT/Pt stacks

One can expect an enhancement of the Gilbert damping in the spin-pumping measurement because additional magnetization damping takes place via “pumping” of the excess angular momentum across the interface into the nonmagnet. On account of the small change of linewidth with scanning frequency, usually the broadband RF signal (10-40 GHz) is required to extract the effective magnetic damping, *α*_eff_:

2 $$\triangle H=\frac{4\pi {\alpha }_{{eff}}}{\gamma }\,f+\triangle {H}_{0}$$

**Fig. 4:**
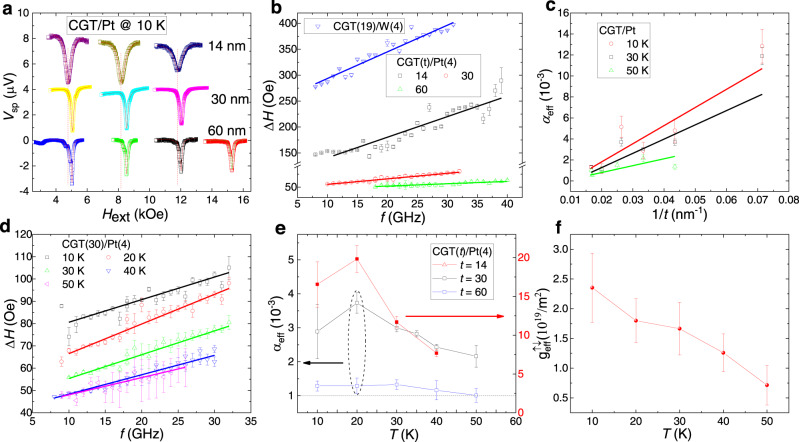
Ultra-low magnetic damping in CGT. **a** *V*_sp_ of CGT/Pt devices with different thicknesses of CGT under a range of RF frequencies to show the frequency and thickness dependence of the linewidth Δ*H*. From the left column to the right column, the frequencies are 11 GHz, 21 GHz, 31 GHz, and 40 GHz, respectively. **b** Linewidth *vs* frequency plotted for different thicknesses of CGT at 30 K, and linear fitting is used to derive the Gilbert damping. **c** Change of effective Gilbert damping with the thickness of CGT is displayed for extracting the contribution of spin pumping in the enhancement of magnetic damping (only the linear regime was used. CGT/Pt devices with *t*_CGT_ thinner than 14 nm and CGT/W are outside the linear zone). **d** Δ*H*-*f* plot for CGT(30)/Pt(4) at different temperatures. **e** Change of effective Gilbert damping with temperature for CGT/Pt with different thicknesses of CGT. **f** Temperature dependence of spin-mixing conductance calculated from the linear fits in (**c**). The solid lines in (**e**) and (**f**) are to guide the eye.

Here, $\triangle {H}_{0}$ is inhomogeneous linewidth. From linear fitting of the frequency-dependent linewidth, $\triangle H$, ${\alpha }_{{eff}}$ is calculated for these stacks with different thicknesses of CGT at different temperatures. Figure 4a shows the spin-pumping signals at different frequencies for the samples with different CGT thicknesses. The Δ*H* was obtained by Lorentzian fitting of *V*_sp_ to extract *α*_eff_ based on Eq. 2 (Fig. 4b, d). An effective magnetic damping constant as low as ~ 1 × 10^−3^ is deduced for the 60 nm-thick CGT at 50 K (see the raw data in Supplementary Fig. S10 and an even lower value ~ (4 ± 1) × 10^−4^ in the sample prepared by the improved sputtering recipe in Supplementary Fig. S6). These values are comparable to that of high-quality YIG thin films at room temperature^45,46^. YIG is a low-damping magnetic insulator. The low magnetic damping in CGT is the parameter that enables the observation of *V*_sp_ with high efficiency. Note a relatively large value of Δ*H*_0_ (30–60 Oe) was found for the CGT/Pt stacks (Fig. 4b), which implies a source of extrinsic damping that may originate from the inhomogeneous effect. For thinner CGT-based devices, assuming the inhomogeneity-induced damping is similar to that of the 60 nm-thick flake, the obvious enhancement of the effective damping (Fig. 4b for the data at 30 K and Supplementary Fig. S11 for that at other temperatures) can only be ascribed to the spin-pumping effect. The effective magnetic damping increases with the decreasing of CGT as predicted by the spin-pumping mechanism^47^:

3 $${{{\alpha }_{{{{{{\rm{eff}}}}}}}={\alpha }_{{eff}}^{{{\infty }}}+\alpha }_{{{{{{\rm{SP}}}}}}}={\alpha }_{{eff}}^{{{\infty }}}+g}_{{eff}}^{\uparrow \downarrow }{\mu }_{B}g/4\pi {M}_{s}t$$

Here, $t$ is the thickness of CGT, ${\mu }_{B}$ is the Bohr magneton, ${\alpha }_{{eff}}^{\infty }$ is the effective magnetic damping of bulk CGT (with *t* → *∞*) and ${\alpha }_{{SP}}$ accounts for the spin-pumping effect. Linearly fitting ${\alpha }_{{eff}}$ versus 1/*t*, we can extract the effective spin mixing conductance, ${g}_{{eff}}^{\uparrow \downarrow }$, for CGT/Pt interface (Fig. 4c). A deviation from linear behavior is found for *t* thinner than 14 nm. The magnetic properties of these thin CGT devices might be severely affected by inhomogeneous strain or the imperfect interfaces. From the fitting of data in the linear zone, a value of ${g}_{{eff}}^{\uparrow \downarrow }$ ~ 2.4 × 10^19^/m^2^ is obtained at 10 K. This value is comparable to that of YIG/Pt at room temperature^48,49^, which reflects its high efficiency of spin angular momentum transmission. Moreover, we find that ${g}_{{eff}}^{\uparrow \downarrow }$ decreases on rising temperature (~ 7 × 10^18^/m^2^ at 50 K, see Fig. 4f). It is distinct from the observation that for various FM/Pt bilayers (the FM can be a metal, a semiconductor, or YIG.) ${g}_{{eff}}^{\uparrow \downarrow }$ does not normally display obvious temperature dependence up to room temperature^37^.

As shown before, the linewidth of the spin-pumping signal also depends on the temperature. For the CGT(30)/Pt(4) sample, *α*_eff_ decreases from 3.7 × 10^−3^ to 2 × 10^−3^ as the temperature increases from 10 to 50 K (Fig. 4e). While *α*_eff_ in CGT(60)/Pt(4) remains almost unchanged below 40 K (although its anisotropy energy *K*_u_ almost doubles at 10 K), it slightly decreases at 50 K. Because of its large thickness, the spin-pumping contribution to *α*_eff_ is not significant in CGT(60)/Pt(4). We speculate that the effective Gilbert damping in bulk CGT is not sensitive to temperatures below 40 K. The obvious increase of *α*_eff_ with decreasing temperature for thinner CGT is mainly due to the larger spin-pumping effect.

## Discussion

One of the advantages of spin-pumping is the generation of coherent spin currents. As key evidence of coherent spin pumping, *V*_sp_ would ideally show no temperature dependence and only depend on the excitation power and rate of magnon relaxation^39,40^. However, coherent spin pumping generated by resonant excitation of magnons (with momentum *k* = 0 for FMR) could be accompanied by the incoherent spin-pumping, i.e., resonant heating causing a temperature gradient and pumping of spin current by the longitudinal spin Seebeck effect (LSSE). In the latter case, the incoherent thermal spin current falls with temperature and vanishes at *T* = 0 K^50^. Therefore, one might qualitatively estimate these two contributions to *V*_sp_ from its temperature dependence. For YIG/Pt, it was reported that the spin-pumping signal rapidly decreased on lowering the temperature below 200 K (see ref. ^40,51,52^, and Supplementary Fig. S9 for the case of YIG(50)/Pt(4) in our control sample). It was questioned whether coherent spin pumping is the main contribution to the observed large spin-pumping signals in the YIG/Pt system at room temperature^40^, although the severe decrease of the spin-pumping signal was also explained as an increase of Gilbert damping in YIG due to impurity relaxation^45,52^. On the contrary, the *V*_sp_ signals in our CGT/Pt system only change slightly (for temperature ≤ 30 K, see Fig. 3b and Supplementary Fig. S9), and we will show that it can be explained by temperature-dependent interfaces properties. It suggests that the CGT offers much larger coherent spin-pumping signals at low temperatures than the thin-film YIG-based system.

Gilbert damping is the intrinsic property of ferromagnetic materials that characterizes the rate of angular momentum dissipation into the lattice. It is the key parameter that determines the critical charge current density in magnetization switching and domain wall propagation speed, as well as spin-wave relaxation^53–55^. In principle, the Gilbert damping of CGT can be measured by the FMR spectra of bulk CGT crystals^56^. However, because of the nonuniformity in the large crystals, their FMR spectra show much larger linewidths (Supplementary Note 2). Here, with the spin-pumping measurement on CGT/Pt devices, a relatively low magnetic damping down to 4-10 × 10^−4^ was found for ferromagnetic insulator CGT (in the presence of spin-pumping), which is the lowest value yet reported in vdW magnetic systems (Table 1 and Supplementary Note 2). It is argued that this value is the upper limit of the intrinsic Gilbert damping in CGT. In addition to the spin-pumping effect, the strain (exerted by the discontinuous Au nanoparticles during exfoliation) and the imperfect interface with the HM thin film can enhance the measured magnetic damping in the form of inhomogeneous magnetization and two-magnon scattering^55^. However, the linear dependence of linewidth on frequency up to 40 GHz in all these devices indicates the minor contribution of two-magnon scattering^54,57,58^ (Supplementary Note 6). Nevertheless, the relatively low Gilbert damping of the CGT flakes, comparable to that of high-quality epitaxial YIG thin films guarantees the potential application of CGT in spintronics, especially when combined with other vdW materials, *e.g*., TMDs and layered topological insulators, for studying the spin-charge interconversion. The low Gilbert damping in CGT might be understood in two ways: the spin relaxation through spin-electron scattering is absent because it is insulating at low temperature; the weak spin-orbit interaction indicated by the tiny orbital magnetization (*g* ≈ 2) avoids the strong spin-phonon scattering^44,59^. Compared with the magnetic damping of *α* ~ 6 × 10^−3^ for a 10.5 nm CGT flake extracted at frequencies of 2–22 GHz in a previous experiment by time-resolved Faraday rotation^59^, we have observed a considerably lower magnetic damping constant in the relatively thicker CGT flakes. The discrepancy is probably related to the (thickness-dependent) crystal quality, and perhaps, more importantly, a wider frequency range (10–40 GHz) is required to extract the damping constant.

**Table 1:** Summary of effective Gilbert damping constant for different materials at different temperature

Materials	Type	Damping factor (10^−3^)	Temperature (K)	Ref.
YIG^a^	Non-vdW-insulator 15 nm	~ 0.9	300 K	^45^
		~ 30	37 K	
YIG^b^	Non-vdW –insulator 65 nm	2.7	300 K	^78^
		1.5	8 K	
Py	Non-vdW –metal ~ 8 nm	8.5	300 K	^79^
Fe_5_GeTe_2_	vdW-metal bulk	35	300 K	^30^
		7	10 K	
CrBr_3_	vdW-insulator bulk	9	30 K	^43^
Cr_2_Ge_2_Te_6_	vdW-insulator ~ 10 nm	6	10 K	^59^
Cr_2_Ge_2_Te_6_	vdW-insulator ~ 60 nm	~1.0	50 K	This work
		1.3	10 K	

**a** and **b** are typical YIG thin films epitaxially grown on Gd_3_Ga_5_O_12_ (111) substrates by off-axis magnetron sputtering and pulsed laser deposition, respectively. Note that the Gilbert damping in high-quality bulk YIG grown by liquid phase epitaxy can be one order of magnitude lower than that in these YIG thin films. However, thin films are more compatible with on-chip electronics devices. All the magnetic damping values in Table 1 were measured without the spin-pumping except in our own work. Note that an even lower magnetic damping value (~4 × 10^−4^) was measured in the samples prepared by the grazing-angle sputtering (Supplementary Note 12).

The spin mixing conductance was also successfully extracted in our work for a hybrid interface with CGT. The values of ${g}_{{eff}}^{\uparrow \downarrow }$ at low temperature are even comparable to the values reported in metallic stacks^37,60^. On one hand, it reminds us that the interface between a vdW ferromagnet and normal metal is not an obstacle for spin transport as the spin angular moments were efficiently delivered across the interfaces in our spin-pumping experiment. It is thus consistent with the reports of highly-efficient spin-orbit torque in CGT/Pt and Fe_3_GeTe_2_/Pt stacks^61–64^. On the other hand, one may attribute the enhancement of ${g}_{{eff}}^{\uparrow \downarrow }$ at low temperature to the to the more ordered interfacial ferromagnetic structures (dashed rectangle regions in Fig. 1f) at the lower temperature, which increase the channels for transferring spin angular momentum of processing magnetic moments in CGT^60,65,66^. We also prepared more batches of samples with controlled interface quality (see Supplementary Note 7). It is found that higher-quality interfaces can give rise to better spin-pumping signals with narrower linewidth and lower effective magnetic damping (spin-mixing conductance can be higher).

However, a larger spin mixing conductance does not always cause larger spin-pumping signals. From Eq. 3 we know that larger ${g}_{{eff}}^{\uparrow \downarrow }$ results in more efficient dissipation of angular momentum from CGT, which in turn increases the system’s effective damping constant (broadening Δ*H*) and hence decreases the precession cone angle $\Theta={h}_{{rf}}/\triangle H$. In Fig. 3b, the fast increase of the amplitude of *V*_sp_ from 70 K to 50 K is the synergy effect of decreasing Δ*H* and increasing ${g}_{{eff}}^{\uparrow \downarrow }$. Below 40 K, although ${g}_{{eff}}^{\uparrow \downarrow }$ keeps increasing, Δ*H* changes to increase as well because of spin-pumping and the PMA enhancement-related inhomogeneity. The temperature dependence of *V*_*sp*_ from 30 K to 10 K shown in Fig. 3b is understandable. This is because *V*_sp_ ~ ${g}_{{eff}}^{\uparrow \downarrow }$/Δ*H*^2^ and ${g}_{{eff}}^{\uparrow \downarrow }$ become 1.4 times larger (Fig. 4f), while Δ*H*^2^ is enhanced 1.5-2 times (see Fig. 3c), thus, one should expect *V*_sp_ at 10 K to be smaller than that at 30 K (the change of resistance and spin diffusion length in Pt are negligible). However, the reality might be more complicated. As we have shown, interfacial diffusion between Pt and CGT is inevitable, which could cause spin memory loss at the interface regions and reduce the amount of spin current detected by ISHE^67^. Another possibility is the magnetic proximity effect of Pt with CGT at low temperature^34^. The effect of temperature-dependent spin memory loss and magnetic proximity effect at the CGT/Pt interface on spin-pumping measurement require further work^68,69^.

We have demonstrated the first spin pumping using a ferromagnetic vdW material, CGT. To the best of our knowledge, the magnetic damping constant, 4–10 × 10^−4^, is the lowest value reported for any vdW ferromagnet. Further work studying high-speed domain-wall propagation and long-distance spin-wave transport is thus anticipated in CGT-based systems. It would be also interesting to study the spin dynamics of CGT in the 2D limit as long as the spin-pumping devices are fabricated with controlled crystal quality and a sharp interface. In addition to the pristine CGT, doped CGT and other magnetic vdW materials with much higher Curie temperature^27,28,30^ and theoretically even lower Gilbert damping^70^ could be studied by spin pumping as well. Our work opens the potential for realizing coherent spin-pumping and even Bose-Einstein condensates of magnons^71^, and superfluid spin current at low temperatures based on CGT and other low-damping magnetic vdW materials^72^. The efficient spin transfer across the vdW gap was revealed by the large spin mixing conductance (2.4 × 10^19^/m^2^) that is even comparable to metallic interfaces. It also makes CGT an important spin/magnon-source building block (without the constraint of epitaxial growth) for spintronic devices (e.g., domain wall racetracks^73^, spin Hall nano-oscillators^74^, magnon transistors) based on the ever-growing family of vdW systems^8,75,76^. A fascinating direction would be investigating the dynamic spin transfer process in the atomically designed sharp interfaces of vdW heterostructures with controllable exchange interaction, magnetic configurations, layer numbers or twist angles. An adequate understanding of the underlying mechanisms (*e.g*., spin memory loss and interface proximity effect) for spin-related momentum and energy transfer across the interfaces could pave the way to versatile 2D spintronics devices.

## Methods

### Preparation of CGT/HM spin-pumping devices

The single-crystal bulk CGT was grown by chemical vapor transport in an evacuated quartz ampule. In order to obtain large-area single-crystal CGT flakes with various thicknesses, an adapted gold-assisted exfoliation method was used^31^. A bilayer of YIG (1.5)/Au (1.5) (nominal thickness ~1.5 nm) was deposited onto clean Si/SiO_2_ wafers by magnetron sputtering. Here YIG (Y_3_Fe_5_O_12-*x*_) is amorphous without ferrimagnetic order and only acts as an insulating seed layer that tightly bonds the SiO_2_ and Au thin films together. Bulk CGT was pre-exfoliated in a glove box (the concentration of O_2_ and H_2_O are below 0.1 ppm) with adhesive tape and stuck the tape with crystals onto the fresh Si/SiO_2_/YIG/Au substrates. Vacuum exfoliation was employed to yield CGT flakes with fresh atomically smooth surfaces and subsequently deposit the HM films with ultra-low sputtering power and relatively high deposition pressure^32^. Only the first 1–2 nm HM was deposited under this milder condition, where representative sputtering angle, ambient Ar pressure, and sputtering power are ~60^o^, ~0.2 Pa, and 10 W, respectively. In the following deposition process, the sputtering power increases step by step, accompanied by decreasing the ambient pressure until reaching the normal sputtering condition (0.08 Pa, 120 W) for the better quality of thin-film HM. We have succeeded in improving the recipe by grazing-angle sputtering at even higher Ar ambient pressure and lower sputtering power to further reduce the damage to CGT (see details in Supplementary Note 3). For the case of CGT/W, additional layers of MgO(2)/W(1) are sputtered after W as protection layers. For the control sample of YIG(50)/Pt(4) used in this work, crystalline YIG (Y_3_Fe_5_O_12_) films were sputtered onto Gd_3_Ga_5_O_12_ (111) single-crystal substrate and annealed at 800-900  ^o^C in air^77^. In the device fabrication process, conventional UV lithography and Ar ion milling were used with special care. Spin coating of additional PMMA ( ~ 100 nm) layer onto the wafers of CGT/HM stacks before the UV resist is important to isolate these stacks from water and oxygen. After UV lithography, the PMMA layer was removed by oxygen plasma shortly before imprinting the patterns onto the films by Ar ion milling. After the first milling process for the CGT/HM slabs (8 µm × (150–380) µm), thick SiO_2_ layer (~80 nm) was sputtered onto the wafers to conformally cover the edges of these thin slabs. After successfully preparing all electrodes with Pt(10)/Au(90), the devices were encapsulated with PMMA again to provide further protection during the measurement.

### Sample characterization

Raman analysis was carried out using a WITec Alpha 300 R system with an excitation wavelength of 532 nm. HAADF-STEM studies were performed in JEM-ARM200 spherical aberration-corrected transmission electron microscope for the Cross-section sample fabricated with a focused ion-beam system.

### Spin-pumping experimental set-up

The spin-pumping measurement was performed in a physical property measurement system (PPMS), Quantum Design, Inc. Microwave was introduced into the PPMS’s chamber by low-loss cable (up to 40 GHz), it was applied on the devices by wire bonding the coplanar waveguide on Logers 4003 chip to GSG pads on the wafer. The voltage signal between the ends of the detection layer was measured by using a lock-in amplifier (SR830).

## Supplementary information

Supplementary Information

## Data Availability

All relevant data are available from the corresponding author on request.
