# Supplementary material for: Electrical detection of spin pumping in van der Waals ferromagnetic Cr2Ge2Te6 with low magnetic damping
Source: Nat Commun. 2023 Jun 28;14:3824. doi: 10.1038/s41467-023-39529-8 (PMC10307887; doi:10.1038/s41467-023-39529-8)
Supplement: Supplementary file 1 — Supplementary Information [file 41467_2023_39529_MOESM1_ESM.pdf]

Supplementary Information

Electrical detection of spin pumping in van der Waals ferromagnetic $Cr_2Ge_2Te_6$ with low magnetic damping

Hongjun Xu[1,2#], Ke Jia[1,3#], Yuan Huang[4#], Fanqi Meng[5], Qinghua Zhang[1], Yu Zhang[1,3], Chen Cheng[1,3], Guibin Lan[1,3], Jing Dong[1,3], Jinwu Wei[1,6], Jiafeng Feng[1,3], Congli He[7], Zhe Yuan[8], Mingliang Zhu[9], Wenqing He[1,3], Caihua Wan[1,2,3], Hongxiang Wei[1], Shouguo Wang[10], Qiming Shao[11], Lin Gu[1,3], Michael Coey[12], Youguo Shi[1,2,3], Guangyu Zhang[1,2,3], Xiufeng Han[1,2,3], and Guoqiang Yu[1,2,3]*

[1]Beijing National Laboratory for Condensed Matter Physics, Institute of Physics, University of Chinese Academy of Sciences, Chinese Academy of Sciences, Beijing 100190, China

[2]Songshan Lake Materials Laboratory, Dongguan, Guangdong 523808, China

[3]Center of Materials Science and Optoelectronics Engineering, University of Chinese Academy of Sciences, Beijing 100049, China

[4]Advanced Research Institute of Multidisciplinary Science, Beijing Institute of Technology, Beijing, 100081, China

[5]State Key Laboratory of New Ceramics and Fine Processing, School of Materials Science and Engineering, Tsinghua University, Beijing 100084, China

[6]Key Laboratory of Magnetism and Magnetic Materials of the Ministry of Education, School of Physical Science and Technology, Lanzhou University, Lanzhou 730000, China

[7]Institute of Advanced Materials, Beijing Normal University, Beijing 100875, China

[8]Department of Physics, Beijing Normal University, Beijing 100875, China

[9]Ningbo Institute of Materials Technology and Engineering, Chinese Academy of Sciences, Ningbo 315201, China

[10]School of Materials Science and Engineering, Anhui University, Hefei, 230601, China

[11]Department of Electronic and Computer Engineering, Hong Kong University of Science and Technology, Kowloon, Hong Kong, China

[12]School of Physics and CRANN, Trinity College, Dublin 2, Ireland

Correspondence to: G.Q. Yu (email: guoqiangyu@iphy.ac.cn)

H.X., K.J., and Y.H. contributed equally to this work.

Supplementary Note 1. Magnetization and uniaxial magnetocrystalline anisotropy constant of bulk $Cr_2Ge_2Te_6$

The single-crystal bulk CGT was grown by chemical vapor transport. Its magnetic property was measured by a vibrating sample magnetometer in PPMS. The measured saturation magnetization at 5 K, M_s ~ 195 emu/c.c., is in good agreement with the values reported in the literature, M_s ~3 μ_B/Cr [1,2]. The magnetocrystalline anisotropy constant of bulk CGT is calculated by the Stoner-Wolfarth model: $2K_u/M_s = H_{sat}$, where H_{sat} is the in-plane saturation field. K_u ~ 4.9×10^5 erg/c.c. was obtained for CGT at 5 K, similar to that in Ref. 2.

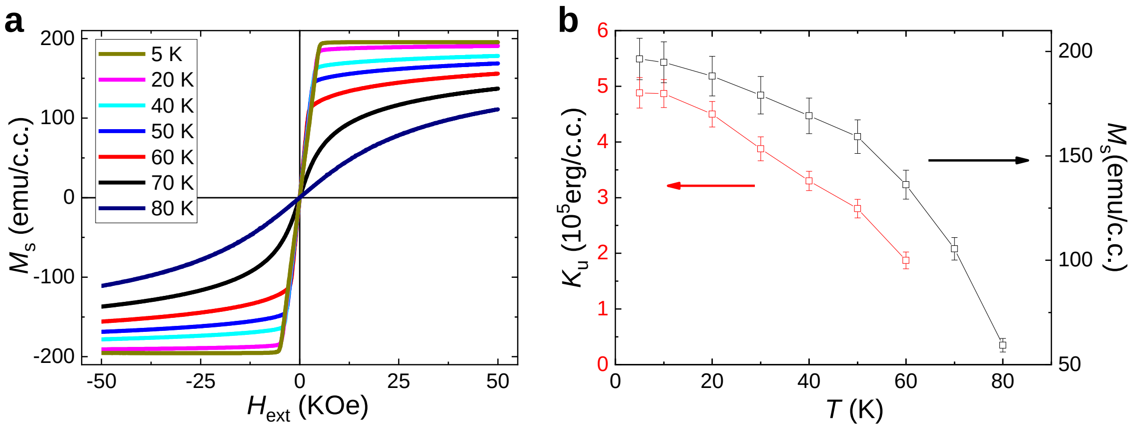

Supplementary Figure S1: Temperature dependence of magnetization (**a**) and uniaxial magnetocrystalline anisotropy constant (**b**) of single-crystal bulk $Cr_2Ge_2Te_6$ used in this work. Error bars throughout this paper correspond to standard errors.

Supplementary Note 2. FMR spectra of bulk CGT crystals

A standard broadband FMR spectroscopy in bulk CGT crystals has been measured by using the FMR spectrometer from NanOsc Instruments AB together with a coplanar waveguide (CPW) placed in a Physical Property Measurement System (PPMS, Quantum Design) system. The bulk crystals were positioned on the signal line of the CPW waveguide. The magnetic field can be applied both along the signal line in the CPW plane (in a-b plane of CGT crystal, in-plane (IP) configuration) or perpendicular to the plane (along the c-axis of CGT, *i.e.*, out-of-plane (OoP) configuration). The typical experimental resonance spectrum measured in CGT crystals with different amounts of weights are shown in Supplementary Fig. S2 **a** & **b**. Both the linewidth and slope of the change of linewidth with frequency are much larger than the results we obtained in spin-pumping devices. For example, the full width at half maximum (FWHM) of bulk CGT crystals is in the range of 600-1200 Oe and $d\Delta H/df$ ~ 10 Oe/GHz. It is found that these values of linewidth are consistent with those reported in the literature[3]. It is believed that these much larger values mainly result from the inhomogeneity of the larger crystals. To verify this statement, we have exfoliated the CGT crystal from a relatively thick (~11.4 mg) crystal to a thin crystal (~0.3 mg) while roughly keeping the area of the crystals the same and measured the FMR spectrum in both IP and OoP configurations respectively. It is found that for these thinner crystals (whose average thickness is still larger than 1 μm) the absorption curves are better fitted by the derivation of a single Lorentzian function. While the distinct deviations from one-peak absorption in the thicker crystals signify the multi-peak absorption (Supplementary Fig. S2 **a**). Not just the linewidth, even the resonance field changed with the thickness (Supplementary Fig. S2 **c**), which reflects the large variations of these related parameters (*e.g.*, multi-crystal domains, crystal magnetic properties,

microwave magnetic field intensity, microwave heating, *etc.*) in both lateral and vertical directions of the crystals. It makes the determination of the intrinsic dynamic properties of bulk CGT inaccurate using this method. However, we can still find some useful information. For all the crystals, including both IP and OoP configurations, the thinner one shows the smaller effective magnetic damping (Supplementary Fig. S2 **d**). The IP and OoP geometry produced roughly similar effective damping ($d\Delta H/df$ ~ 7.7-8.7 Oe/GHz for the mid-thick CGT crystal ~1.2 mg), but the former displayed smaller linewidth. Hence, the values measured by the IP geometry FMR of the thinnest crystal (~0.3 mg) should be closer to the lowest intrinsic Gilbert damping constant value in CGT (Supplementary Fig. S2**b**). Actually, the slope of frequency dependence of the linewidth of that CGT crystal is quite small, and a linear fitting gives rise to a magnetic damping ~$1.1 \pm 0.9 \times 10^{-3}$, which already approaches the lowest values measured by the spin-pumping. Improving the homogeneity of the crystals and enhancing the accuracy of the measuring system should further lower this value. We note that in literature the linewidth of electron spin resonance spectroscopy in bulk CGT single crystal changed slowly over a wide frequency range[4], *i.e.*, from ~281 Oe at 32.4 GHz to ~356 Oe at 271.1 GHz, linearly fitting of this range leading to a Gilbert damping constant ~9×10^{-4}, which is consistent with that of our spin-pumping measurement. However, our spin-pumping measurement displays better performance than the bulk FMR experiments in terms of much narrower linewidth and homogeneity broaden and better signal-to-noise ratio for extracting the magnetic damping parameters. It highlights that spin-pumping or ST-FMR measurements on the magnetic vdW systems with smaller sample volume and (therefore) better homogeneity is necessary to unveil any intrinsic ultra-low damping systems which could be otherwise concealed by the conventional bulk FMR measurement.

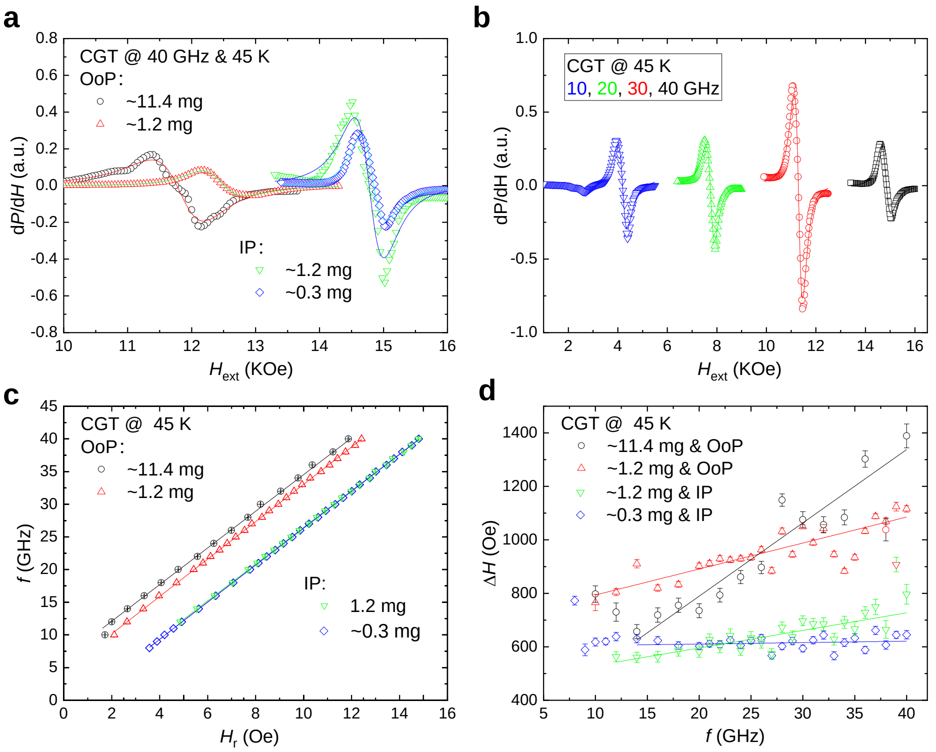

Supplementary Figure S2. (**a**) typical FMR spectra for CGT crystals with different thicknesses (*i.e.*, weights) and under IP and OoP detection geometries. (**b**) frequency dependence of FMR spectra of the thinnest CGT (~0.3 mg) under IP configuration. *f*-*H* (**c**) and Δ*H*-*f* (**d**) curves for these samples at 45 K. The data in (**a**) and (**b**) are fitted by the derivation of a single Lorentzian function, while the points in (**c**) and (**d**) are fitted by Kittel's equation and straight lines, respectively.

Supplementary Note 3 Improved sputtering recipe for better spin-pumping devices

Grazing-angle sputtering with increased Ar ambient background pressure and decreased sputtering power (*i.e.*, acceleration voltage) could greatly release the high energy of sputtered atoms and reduce the damage to 2D materials[5]. The representative sputtering angle, Ar gas pressure, and sputtering power are ~0°, ~1 Pa, and 2-3 W for the grazing-angle sputtering, respectively (see the schematic illusion in Supplementary Fig. S3a). For the preparation of CGT/Pt bilayer structures, only the first ~1 nm Pt was deposited by grazing-angle sputtering (rate ~ 0.1 nm/min) while keeping the substrate

stationary. The substrate is rotating in the following deposition process, which changes the sputtering angle between ~0° and ~60°, and the sputtering power increases step by step, accompanied by decreasing ambient pressure until reaching the normal sputtering condition (0.08 Pa, 120 W). By doing this, we find the quality of CGT/Pt interfaces is improved, as evidenced by the enhanced intensity of Raman spectra in the ultra-thin CGT/Pt bilayer (even the monolayer MoS_2 can preserve its Raman spectrum after the grazing-angle sputtering of Pt, see Supplementary Fig. S3b). More importantly, we find that both the inhomogeneity broadening and effective magnetic damping of CGT/Pt were reduced. For the CGT crystals of ~60 nm covered by nominated 4nm-thick Pt, the ΔH_0 is decreased from ~44 Oe to ~36 Oe and the magnetic damping factor from ~ 10.4×10^{-4} to ~ 9×10^{-4} (Supplementary Fig. S3 c&d).

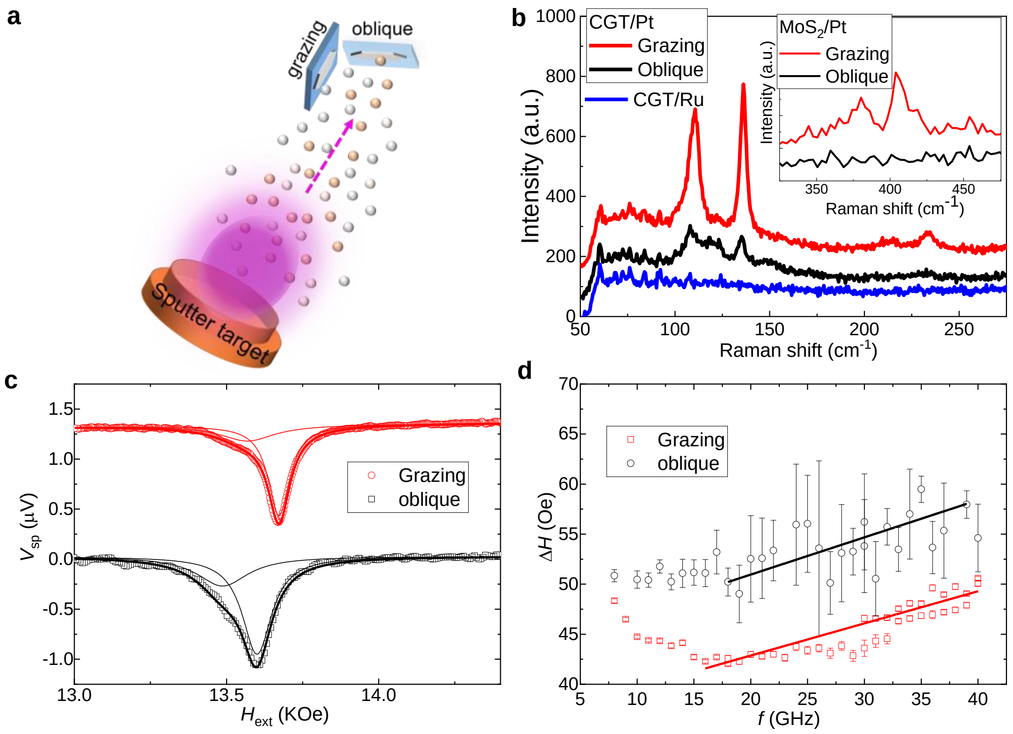

Supplementary Figure S3. (**a**) the schematic diagram of oblique-angle sputtering and grazing-angle sputtering techniques. (**b**) Representative Raman spectra of ultra-thin CGT after sputtering metals with different recipes. Inset shows the Raman spectra of monolayer MoS_2 after sputtering Pt. (**c**) Typical spin-pumping signals at 36 GHz (with

two-peaks fitting) and frequency dependence linewidth (**d**) of these devices based on relatively thick CGT crystals (~60 nm) prepared by different recipes at 40 K.

Supplementary Note 4. Anomalous Hall effect and anisotropic magnetoresistance in CGT/Pt and CGT/W.

Because CGT is insulating at low temperatures, its resistance contribution to the electrical signal in CGT/HM is negligible, and the longitudinal and transverse resistances can only be generated by the HM with possible magnetic proximity effect or spin Hall magnetoresistance. Anomalous Hall effect (AHE) on CGT(6)/Pt(4) and CGT(8)/W(4) are shown in Supplementary Figures 4 **a** & **b,** respectively. In contrast to the soft perpendicular magnetization displayed in pristine CGT flakes detected by Kerr rotation[6], clear coercivity emerges in the thin CGT flakes with the proximity of the HM capping layer, which indicates the enhanced perpendicular magnetic anisotropy because of the HM. AHE signals vanished at ~ 70 K, which reflects a Curie temperature around this value. An obvious non-linear behavior was found in the R_H-H_z curve of CGT/W stacks for a temperature above the Curie temperature of CGT (*e.g.*, at 80 K shown in yellow curve in Supplementary Fig. S4 b), which might be related to the multi-bands conduction in the inter-diffusion interface region of CGT/W. Anisotropic magnetoresistance (AMR) of CGT/Pt was measured by rotating the in-plane field ($\mu_0 H$ = 1 T). The angular dependence of R_{xx} is fitted by $R_A*\cos^2\varphi + R_B*\cos\varphi$ (the solid curves in **c**). Here, R_A and R_B present the contributions of AMR and the background of Hall signals, respectively. The extracted temperature dependence of AMR contribution is plotted in **d.** It is observed that the AMR ratio ($\leq 2 \times 10^{-5}$) in CGT(30)/Pt(4) is considerably lower than that in YIG/Pt ($\sim 10^{-4}$)[7], which means the spin rectification

signals of CGT/Pt in spin-pumping measurement should be even smaller than that in YIG/Pt. Similarly, it disappeared at ~ 70 K, consistent with the results of AHE.

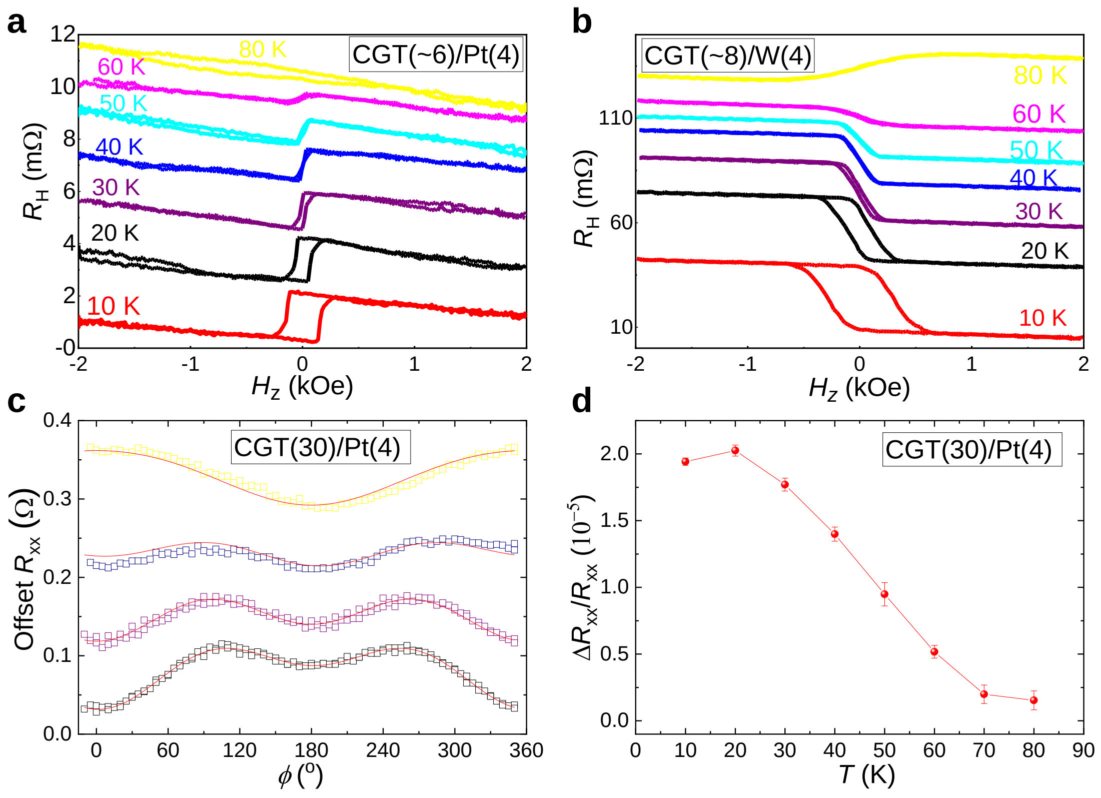

Supplementary Figure S4. Anomalous Hall effect and anisotropic magnetoresistance in CGT/HM stacks. Temperature dependence of anomalous Hall effect in CGT(6)/Pt(4) (**a**) and CGT(8)/Pt(4) (**b**). (**c**) Angular dependence of magnetoresistance of CGT(30)/Pt(4) and its fitting. From bottom to top, the data are measured at 10, 30, 50, and 70 K, respectively. The extracted temperature dependence AMR ratio is shown in (**d**).

Supplementary Note 5: Multiple resonant peaks of thicker CGT devices

Multiple peaks are frequently observed for the devices with thicker CGT flakes (≥ 30 nm). For CGT(60)/Pt(4), its multiple peaks structure comprises two series of peaks. The

first series of peaks, multi-domain ferromagnetic resonance mode[8], occurs when the field applied is smaller than the in-plane saturation field (~4000 Oe at 10 K), where the incline background signal changes into the flattened one. The resonant peaks in multi-domain modes show broader and weaker peaks than the uniform mode of FMR. This mode was only observed in the sample with the thickest CGT in this work. The second series of peaks show up as the satellite peaks of the main peaks at the larger in-plane field. An example of the profile fitting of this series with 4 peaks is shown in Supplementary Figure 5 **a.** It is speculated that these peaks are generated by the surface and bulk modes of standing waves in the relatively thick CGT[9] or related to the inhomogeneity of CGT. The detailed identification of them needs further study but is beyond the scope of this work. We have performed the multiple Lorentzian function fitting for all the data which emerged more than one peak. A summary of f-H_r for CGT(60)/Pt(4) at 10 K is displayed in Supplementary Fig. S5 **b & c**. The devices with thick CGT usually exhibit multi-peak signals, and they are dominated by the main peaks. Linewidth extracted by multi-peak fitting can sometimes yield relatively large error margin due to a number of fitting parameters. Normally, the linear relationship between linewidth and frequency for the main peak is obvious when the resonant field is larger than the in-plane saturation field, while for these satellite peaks, the linear relationships between linewidth and frequency are not as obvious as that of the main peak (Fig. S5 **d**). The relatively large perpendicular magnetic anisotropy of CGT together with the non-zero tilted angle ($< 10^o$) of the external field with the *a*-*b* plane of CGT can give rise to the increase of linewidth with decreasing frequency at the lower frequency range because of the field drag effect[10,11].

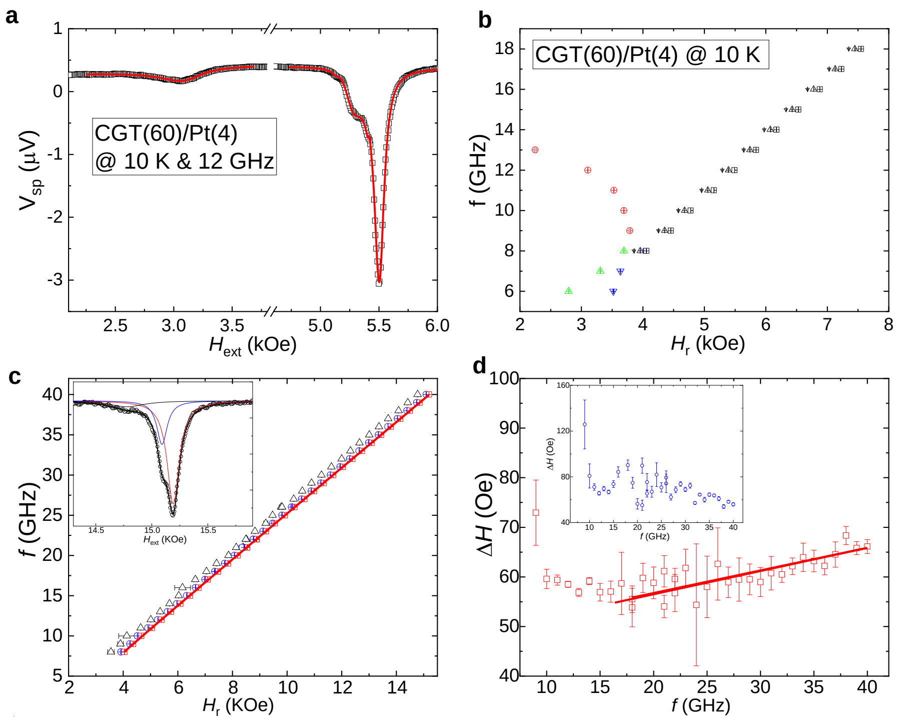

Supplementary Figure S5. Multiple resonant peaks in the device of CGT(60)/Pt (4) at 10 K. (**a**) It comprises two series of peaks depending on the magnitude of resonant field. (**b**) The summary of *f*-*H*r at different field ranges, where the red dots mark the multi-domain modes, the green and blue dots are probably related to the domain wall resonance mode[8], and the black square and triangles present the main peak and satellite peaks respectively. (**c**) *f*-*H*r plots of these different peaks which are obtained by the three-peak fitting (the example of 40 GHz is shown in the inset). (**d**) frequency dependence of ΔH of the main peak and the secondary peak (inset) after three-peak fitting.

Supplementary Note 6. Elimination of two-magnon scattering

We have further performed some experiments to verify the reliability of our experimental configuration and eliminate the contribution of two-magnon scattering. We studied the second experimental geometry by making the CGT/Pt slab right underneath the signal line of CPW (with ~150 nm SiO_2 between them) to reduce the possible effect of inhomogeneous RF field in previous spin-pumping configuration (see Supplementary Fig. S6a)[12]. We note that the amplitude of spin-pumping signals in this new configuration is visibly smaller than the first configuration used for the devices in

main text under the same power of RF source signal, which is partly due to the geometry correction factor of $\cos^2\alpha\sin\alpha$, where $\alpha \approx 45^o$ is the angle between the external magnetic field and the longitudinal direction of the slab[13]. Nevertheless, we managed to measure the spin-pumping signals up to 40 GHz (the highest frequency we can reach) for the devices with thick CGT crystals. As shown in Supplementary Fig. **S6c** for the sample of CGT(45)/Pt(4), the obvious linear dependence of linewidth on frequency is observed in the frequency region of 10 to 40 GHz, and the effective magnetic damping calculated from the slope of $d\Delta H/df$ is in the range of 9-12 × 10^{-4}. It nicely reproduces the results of our devices measured in precious detection configuration and proves that the results obtained from our previous experimental configuration are authentic. Moreover, based on the linear behavior in the data of ΔH-f, we can actually exclude the significant effect of two-magnon scattering as it normally shows a steep nonlinear slope at low frequencies and saturates at high frequencies[14-16]. Note that even for the control samples of Pt/Au/CGT, which own apparent rougher interfaces and potential higher contribution of two-magnon scattering, the typical non-linear behavior of two-magnon scattering is not observable (blue data points in Supplementary Fig. S6c). It further indicates the two-magnon scattering can be ignored in our system.

On the other hand, if two-magnon scattering exists in CGT and the scattering centers in CGT are given by lattice defects, the angular dependence should reflect this lattice symmetry, and we might expect an angular dependence of effective magnetic damping depending on the magnetization direction relative to the crystallographic direction[17,18]. For a given spin-pumping device, this angular-dependent damping can be measured by applying the magnetic field in different directions. We thus fabricated a new device with thicker CGT (~80 nm) covered by a slightly thinner Pt with high-quality interface (deposited by grazing-angle sputtering, thickness ~3 nm, length of spin detector ~380 μm) to guarantee the spin-pumping signals are always large enough even when the magnetic field largely deviates from the direction with the maximal signals ($V_{sp} \propto \sin\Phi$, Φ is the angle between the magnetic field and the longitudinal direction of the spin detector). The representative results are displayed in Fig. S7d for the spin-pumping signals at 40 GHz (note that the RF power applied in the measurement of Φ ~47° is larger than these in the other angles). The high-quality data enable us to perform the multi-peak fitting for each frequency at different angles. The results of the main peak extracted from the multi-peak fitting are shown in Figs. S6 e & f. Both the f-H_r and ΔH-

f plots present very few differences at different angles. Although there is an offset between the data point measured at ~47° and others, the slopes of them remain unchanged in the error level of our measurement (~0.15 ± 0.03 Oe/GHz). Therefore, the absence of angular dependence in these effective magnetic damping under different magnetization directions reconfirms the negligible two-magnon scattering in our CGT/Pt system once again. Here, we obtain the lowest effective magnetic damping of CGT in this work, *i.e.*, ~ (4±1) ×10^{-4}.

Furthermore, since the linewidth of the main peak of the sample shown in Fig. S6d at 40 GHz is ~45 Oe (mainly consisting of the inhomogeneous broaden), we can use this value to roughly calculate the upper limit of the Gilbert damping in CGT (from dΔH/df ~ 1.1 Oe/GHz), ~ 3 ×10^{-3}. If we use the linewidth of the secondary peak ~20 Oe at 40 GHz and an even smaller upper limit of the Gilbert damping (~ 1.3 ×10^{-3}) in CGT can be obtained, which is consistent with our results deduced by linear fitting of the ΔH-f data points.

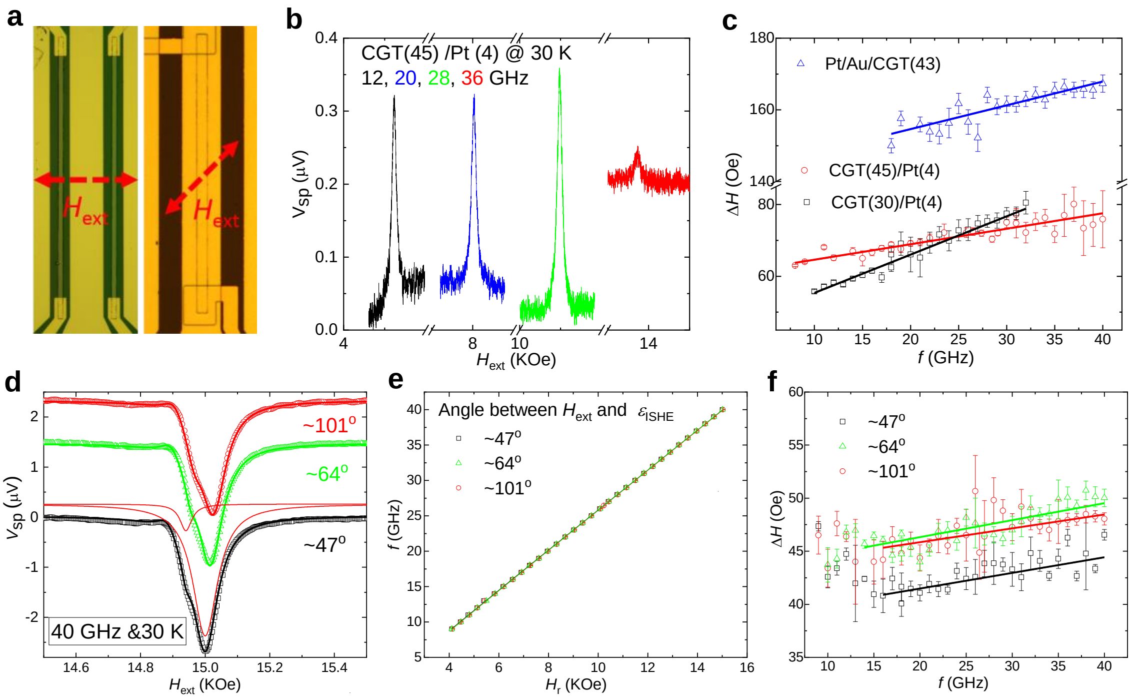

Supplementary Figure S6. (**a**) optical microscopic images for spin-pumping devices fabricated with different geometries. The left (right) one is the first- configuration (second-configuration) device with the in-plane external magnetic field applied at an angle of ~90° (45°) with both the CGT/Pt slab and the microwave magnetic field. (**b**) Typical spin-pumping signals the second-configuration devices, CGT(45)/Pt(4) at 30

K. (**c**) Comparison of linewidths of the spin-pumping devices fabricated with different geometries at 30 K, where the data measured under the first configuration is plotted by by black and blue patterns and the second configuration by red circles. (**d**) Spin-pumping signals of the CGT(80)/Pt(3) stacks at 30 K and their two-peak fitting with two symmetric Lorentzian peaks. The frequency dependences of H_r and ΔH of the main peak are plotted in (**e**) and (**f**), respectively, for different angles. Data in (**c**) and (**f**) are linearly fitted for calculating the effective magnetic damping.

Supplementary Note 7 spin-pumping in control samples

In order to preliminarily investigate the effect of the ill-defined interface, we have prepared new batches of spin-pumping devices with different interface properties. For the batch of CGT/Pt prepared by the grazing-angle sputtering process, their interfaces are of the highest quality as characterized by the Raman spectra (Supplementary Fig. S3b). We found the effective magnetic damping ($\alpha_{eff} \sim 8\times10^{-4}$) with a relatively small inhomogeneity broadening (~45 Oe) in the CGT(60)/Pt at 30 K. The spin-mixing conductance in those batches of samples can be derived by the linear fitting of α_{eff} with $1/t_{CGT}$. The value ~$2.4 \pm 0.7 \times 10^{19}/m^2$ is also larger than that of the samples prepared by the oblique-sputtering process (~$1.7 \pm 0.5 \times 10^{19}/m^2$, see Supplementary Fig. S7c). It is consistent with our assumption that the ordered interface can pump out of the spin angular momentum more efficiently. It also agrees with the ab initio calculation that the abrupt interface favors less spin memory loss[19]. Another control sample is CGT/Ru, where the Ru layer is sputtered at a condition that is harsh for CGT: oblique-angle sputtering, 0.08 Pa, and 50 W. The interface is ill-defined, and several layers of CGT can be damaged (as the characteristic Raman spectra of CGT is undetectable in the stacks with ultra-thin CGT crystals, see Supplementary Fig. S3b). Weak and broader spin-pumping peaks were only detectable in the devices with thick CGT, as displayed in Supplementary Fig. S7a for CGT(75)/Ru(4), and the effective magnetic damping (~9×10^{-3} at 30 K) is one order of magnitude larger than the CGT(60)/Pt prepared at a milder condition. Here the larger effective magnetic damping enhancement can be understood as the enhanced spin-memory loss at the ill-defined interface region, considering that Ru is not an efficient spin sink as Pt[20] (and it shows no magnetic proximity effect). Additionally, we have succeeded in directly exfoliating the CGT crystal onto the Pt(3)/Au(1.0) bilayer. For the Pt/Au/CGT devices, although the damage

to the interface by sputtering and the magnetic proximity effect of CGT on Pt can be avoided, the discontinuous Au particles of nanometer size significantly increase the interface roughness compared with CGT/Pt devices (as only parts of CGT bottom surface tightly touch the Pt/Au), the ΔH_0 clearly enhanced compared with CGT/Pt devices while the effective damping is slightly increased (Supplementary Fig. S7b).

From our control experiments, we believe that a high-quality interface is necessary to access the intrinsic properties of magnetic vdW materials in terms of Gilbert damping and spin-mixing conductance.

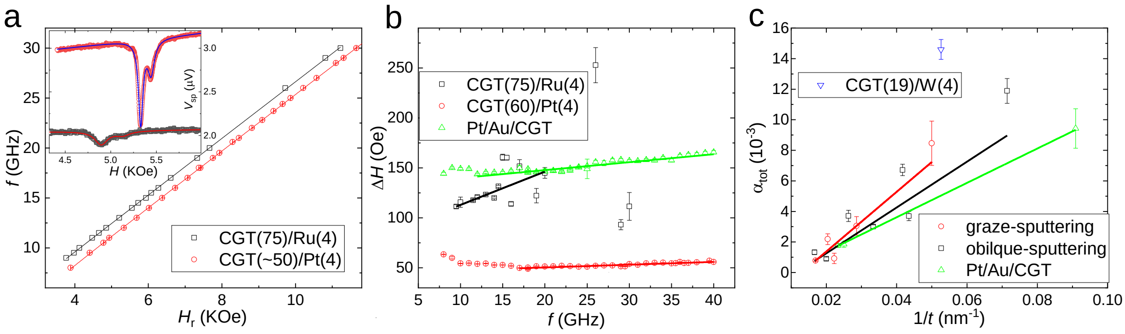

Supplementary Figure S8. Control experiments at 30 K. (**a**) f-H_r plots for CGT(50)/Pt(4) and CGT(50)/Ru(4), both of them were fabricated with the new configuration. The inset of (**a**) shows their typical spin-pumping signals at 12 GHz. (**b**) frequency dependence of linewidth of the representative control samples (**d**) α_{eff} *vs.* 1/t for the CGT/Pt prepared by different recipes.

Supplementary Note 8. spin-pumping signals in the thinnest and thickest CGT-based devices.

Spin-pumping signals of CGT/Pt were detectable for the thickness of CGT is larger than 5 nm, and at the temperature below ~75 K. The examples of the V_{sp} measured at the extreme conditions of this work are shown in Supplementary Fig. S8 for CGT(6)/Pt(4) at 30 K (**a**, **b**, **c**) and CGT(60)/Pt(4) at 70 K (**d**, **e**, **f**). Although these signals are only obvious in the limited range of field, M_{eff} can be extracted from the

fitting of data points in the f-H_r plot, while the linear fitting of ΔH *vs.* f is not good enough because of the low signal-noise ratios. In spite of this, our spin pumping measurement has approached the experimental limitations when the temperature is close to the Curie temperature of CGT or the thickness of CGT is in the atomically thick range, which actually presents the state-of-the-art level in the similar measurement of dynamic magnetic properties in the vdW system.

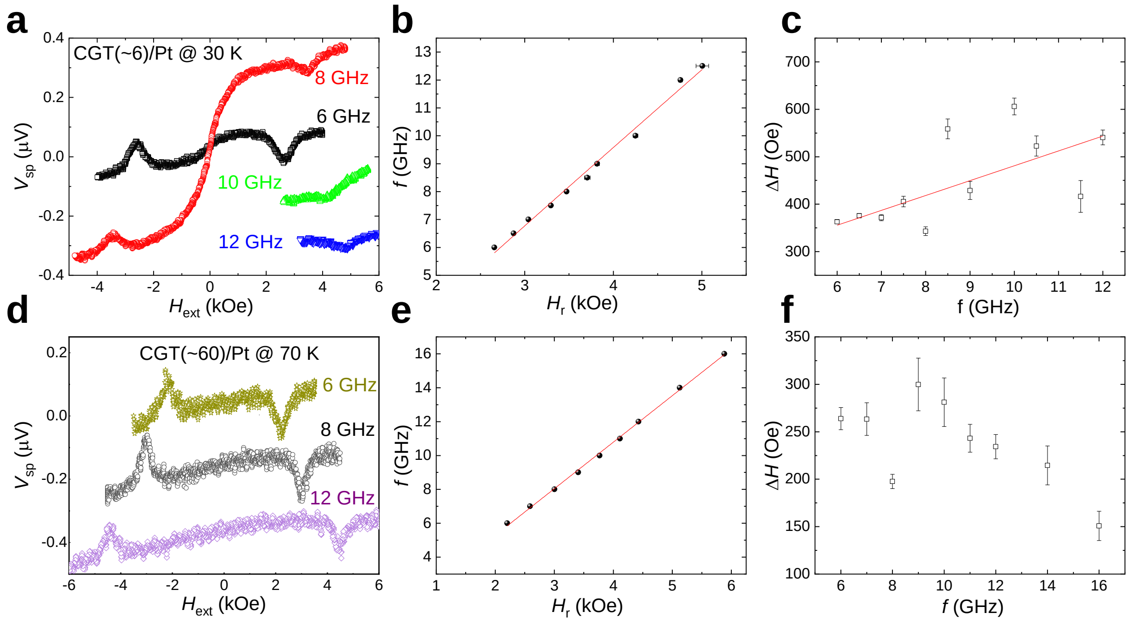

Supplementary Figure S8. V_{sp} measured in extreme conditions. Frequency dependence of V_{sp} of CGT(6)/Pt(4) at 30 K (**a**), from the Lorentzian fitting of these peaks, the frequency dependence of H_r and linewidth are displayed in (**c**) and (**d**), respectively. Similar results for CGT(60)/Pt(4) at 70 K are shown in (**d**), (**e**) and (**f**).

Supplementary Note 9. Temperature dependence of V_{sp} in CGT/Pt and YIG/Pt.

Temperature dependence of V_{sp} under the excitation 28 GHz RF signal at 10-60 K shows the same trend as that of V_{sp} at 12 GHz for stacks of CGT(30)/Pt(4). These two RF signals are amplified by two different amplifiers working at different frequency ranges, which are compared to exclude the possible influence of frequency-dependent microwave loss. The V_{sp} firstly increases with lowering the temperature until reaching

a maximum at ~ 30 K and then slightly decreases. The dependence can be well reproduced in different samples (see the results of CGT(23)/Pt(4) in Supplementary Fig. S9 **b** and **c**) under different frequencies. This temperature dependence is determined by the inherent properties of CGT/Pt, including magnetization, effective magnetic damping, spin mixing conductance, *etc*.

It is worth comparing the temperature dependence of V_{sp} in CGT/Pt with that in YIG/Pt, which is a well-studied spin-pumping system. These YIG thin films were epitaxially grown on $Gd_3Ga_5O_{12}$ (111) substrate by magnetron sputtering. The YIG(50)/Pt(4) bilayer devices were fabricated with the same dimension as CGT/Pt devices and measured in the same system. As shown in Supplementary Fig. S9 **d-f**, the V_{sp} dramatically decreased with temperature, with the linewidth significantly increased. This behavior is in agreement with that reported in much thick and high-quality YIG (thickness ~200 μm) in Ref.[9], which suggested the inherent properties of YIG films at low temperatures. Its strikingly enhanced magnetic damping was also ascribed to the strong magnetic relaxation via rare-earth ions[21]. Because the signals in **a** and **d** were excited by RF sources of −17 dBm and −12 dBm, respectively, we can expect that the V_{sp} in CGT(30)/Pt(4) is 1-2 orders of magnitude larger than that in YIG(50)/Pt(4) under the same condition when the temperature is below 50 K. From the control experiment, we confirmed the fidelity of the measuring system and demonstrated the much higher spin-pumping efficiency of CGT/Pt at low temperatures than that of thin-film YIG/Pt. Moreover, V_{sp} in CGT/Pt (≤ 50 K) is relatively insensitive to the temperature, which suggests the coherent spin-pumping in the system.

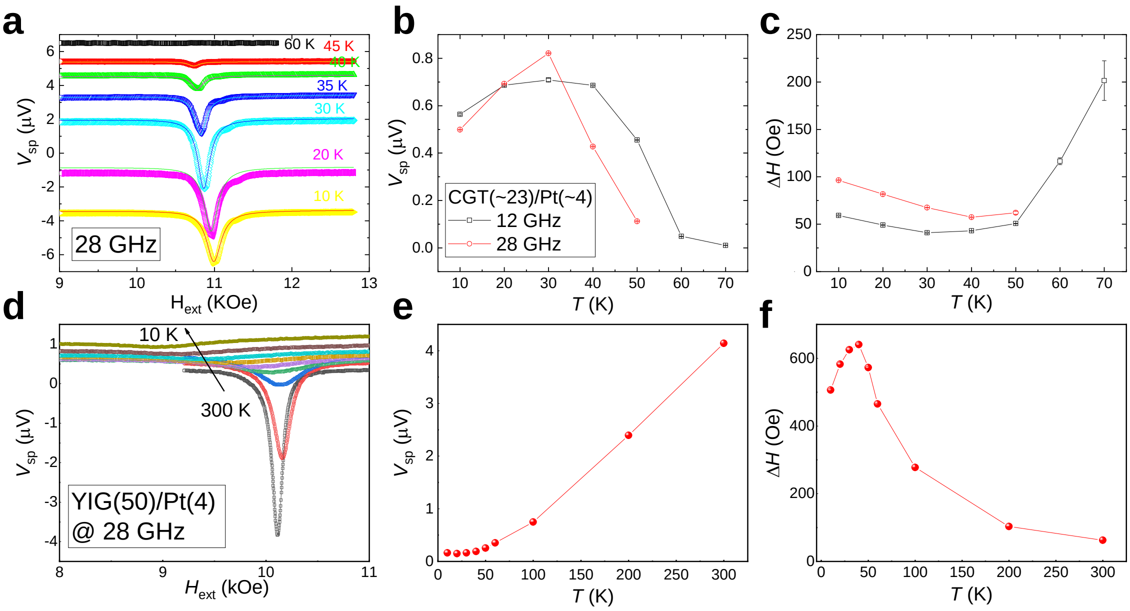

Supplementary Figure S9. Temperature dependence of V_{sp} in CGT/Pt and YIG/Pt. Temperature dependence of V_{sp} under the excitation 28 GHz RF signal for CGT(30)/Pt(4) (**a**) and YIG(50)/Pt(4) (**d**). A similar trend of the temperature dependence of V_{sp} (**b**) and ΔH (**c**) are observed for CGT(23)/Pt(4), which are distinct from that observed in YIG(50)/Pt(4) (e) and (**f**).

Supplementary Note 10. Temperature dependence of effective magnetic damping for 14 nm- & 60-nm CGT.

CGT(60)/Pt(4) stack possesses one of the lowest effective magnetic damping values at 50 K in our measurement (of the samples prepared by oblique sputtering). The corresponding frequency dependence of V_{sp} is displayed (in Supplementary Fig. S10 **a** & **b**). Their background (off-resonant) signals markedly decreased compared with that at lower temperatures. The temperature dependence of effective magnetic damping for CGT(60)/Pt(4) and CGT(14)/Pt(4) can be extracted from the slopes in ΔH-f plot (Supplementary Fig. S10 **c** & **d**). It is found that for almost all the stacks with relatively thick CGT flakes, their effective magnetic damping tends to decrease as temperature increases up to the range of 40-50 K. The underlying mechanisms of the notable

temperature dependence of effective magnetic damping and spin mixing conductance require further investigations.

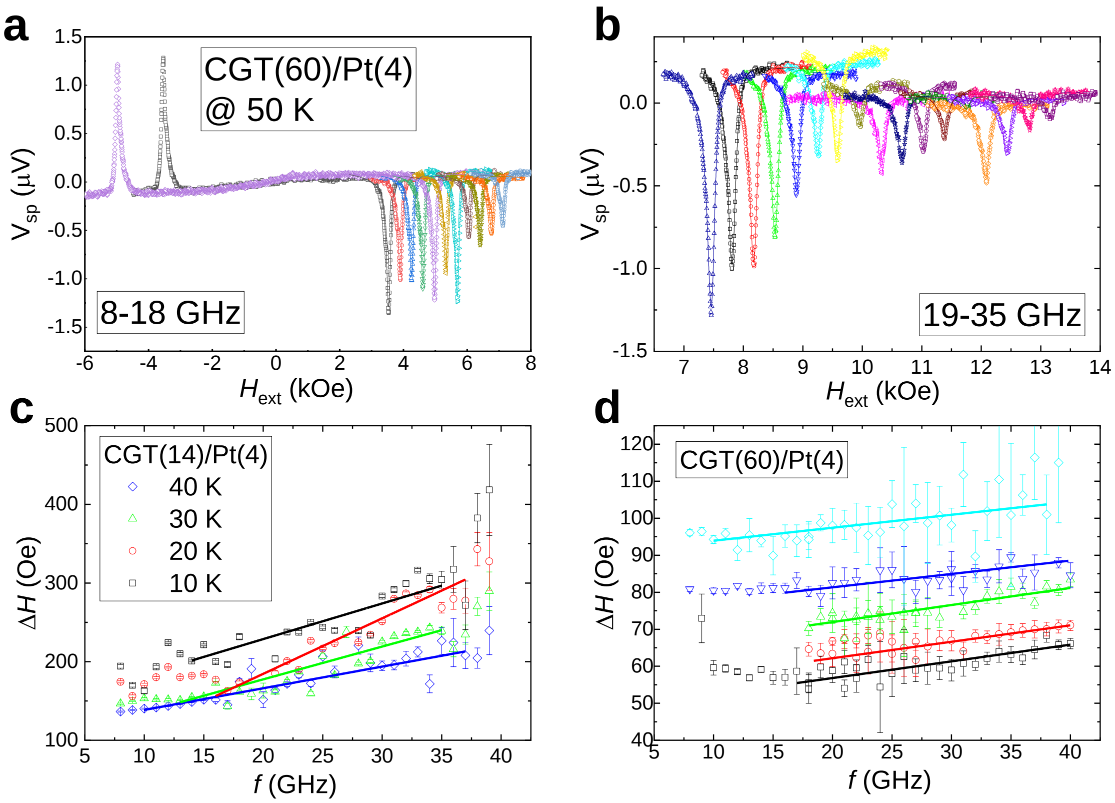

Supplementary Figure S10. V_{sp} of CGT(60)/Pt(4) under the frequencies of 8-35 GHz (**a & b**) at 50 K. Temperature dependence of magnetic damping in CGT(14)/Pt(4) (**c**) and CGT(60)/Pt(4) (**d**). Note that the data points of different temperatures (from bottom to top corresponding to 10, 20, 30, 40, 50 K respectively) in (**d**) are vertically shifted.

Supplementary Note 11. Thickness dependence of magnetic damping at 10, 20, 40, and 50 K.

The linewidths as the function of frequency are plotted for CGT/HM at 10, 20, 40, and 50 K for different thicknesses of CGT are displayed in Supplementary Fig. S11 **a-d**. It is clear that in the stacks of CGT/Pt, the device with the thickest CGT shows the smallest slope, *i.e.*, effective magnetic damping, as expected from the spin-pumping

effect. The slopes tend to decrease as temperature increases. However, only for the sample with CGT flakes thick enough (> 20 nm), their effective magnetic damping can be reliably calculated by linear fitting in Δ*H*-*f* plot at 50 K.

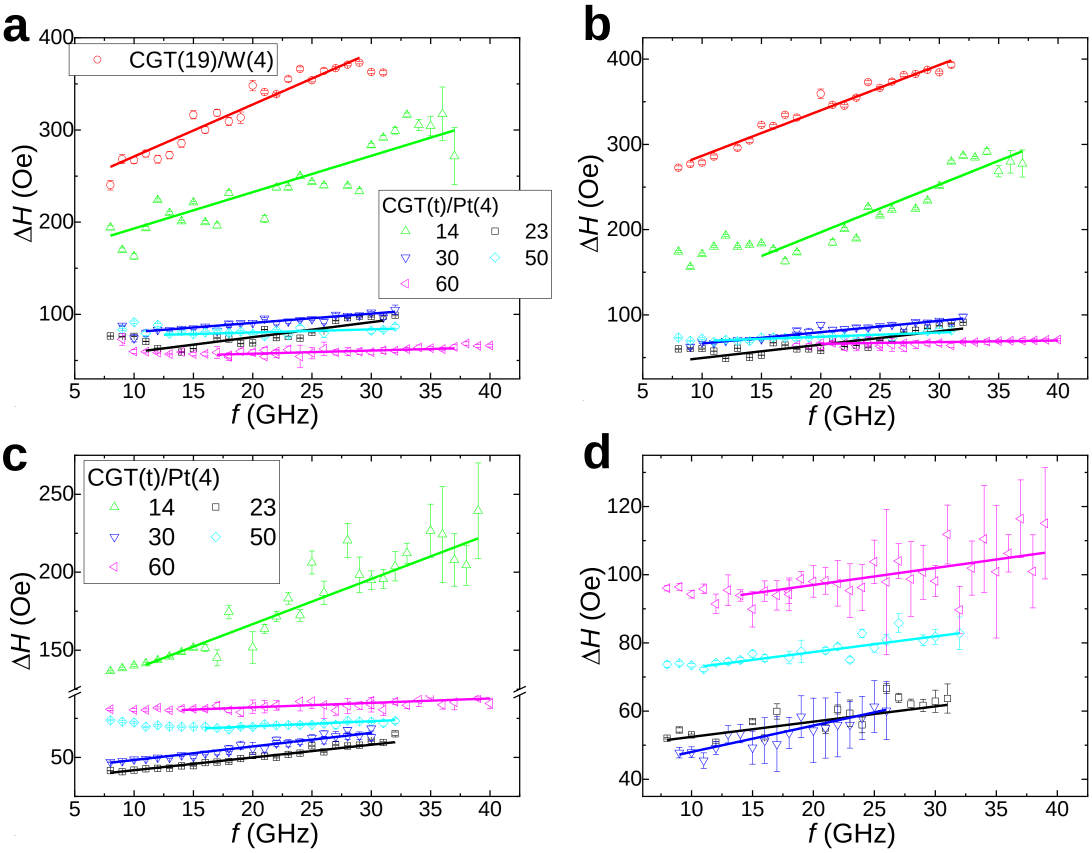

Supplementary Figure S11. Frequency dependence of linewidth for the devices (prepared by oblique sputtering) with different CGT thicknesses at 10 (**a**), 20 (**b**), 40 (**c**), and 50 K (**d**).

Supplementary Note 12. The summary of spin-pumping devices fabricated in different batches at 30 K.

The extensive spin-pumping measurements were performed at 30 K where the devices normally displayed the largest signals. The lowest effective magnetic damping constant that is below 1×10^{-3} (even down to 4×10^{-4} in our results) was repeated in different samples prepared with different recipes. It actually reflects the intrinsic Gilbert damping of the CGT should be below 1×10^{-3}. Although these are some fluctuations in the thickness dependence of effective magnetic damping, we can still find that it increases

with the decrease of thickness, which manifests the spin-pumping effect. The spin-mixing conductance of the CGT/Pt system was thus estimated as ~ $2 \times 10^{19}/m^2$ at 30 K depending on the quality of the interfaces.

Table 1. The summary of spin-pumping devices fabricated in different batches, where the magnetic properties are extracted by the fitting of spin-pumping data (including the f-H_r and ΔH-f plots). The series numbers denote the different fabrication recipes and measurement configurations with varied thicknesses. A 4 nm-thick Pt electrode was used in most of the devices as the spin detector, excepting the cases with specific annotations.

Sample's number	Thickness of CGT (nm)	$\mu_0 M_{eff}$ (mT)	H_0 (Oe)	α_{tot} (10^{-3})	Condition (@ 30 K)
#1-1	~6	-102	167.8	88 $\pm$ 22	Oblique sputtering
#1-2	14	-132	91.5	11.7 $\pm$ 0.7	Oblique sputtering
#1-3	19	-111	242.2	14.4 $\pm$ 0.6	W, Oblique sputtering
#1-4	23	-156	28.3	3.7 $\pm$ 0.3	Oblique sputtering
#1-5	24	-59.2	104.2	6.7$\pm$ 0.4	Oblique sputtering
#1-6	30	-175.4	44.7	3.0 $\pm$ 0.1	Oblique sputtering
#1-7	38	-127.8	93.9	3.7 $\pm$ 0.4	Oblique sputtering
#1-8	~50	-145.8	61.1	0.9 $\pm$ 0.1	Oblique sputtering
#1-9	60	-179.4	42.5	1.3 $\pm$ 0.1	Oblique sputtering
#2-1	16	-207.5	115.2	3.5$\pm$ 0.6	Graze sputtering
#2-2	35	-188.7	65.3	3.1$\pm$ 0.6	Graze sputtering
#2-3	59	-186.5	44.8	0.8$\pm$ 0.1	Graze sputtering
#2-4	20	-163	113	8.5$\pm$ 1.4	45°, Graze sputtering
#2-5	21	-177.1	120	3.5$\pm$ 0.8	45°, Graze sputtering
#2-6	45	-177.6	62.9	0.9$\pm$ 0.3	45°, Graze sputtering
#2-7	49	-215.3	26.1	2.6 $\pm$ 0.1	45°, Graze sputtering
#3-1	75	-112.7	79.7	9.3 $\pm$ 4.6	Ru, normal sputtering
#4-1	12	-113.7	254	9.4 $\pm$ 1.3	Pt/Au/CGT
#4-2	43	-130.3	190	1.0 $\pm$ 0.2	Pt/Au/CGT

#4-3	40	-171.7	141.4	1.8 ± 0.2	Pt/Au/CGT
#5-1	24	-166.4	69.9	2.0 ± 0.2	Pt ~ 3 nm, Graze sputtering
#5-2	80	-165.1	43.5	0.4 ± 0.1	Pt ~ 3 nm, Graze sputtering

References

1 Ji, H. *et al.* A ferromagnetic insulating substrate for the epitaxial growth of topological insulators. *J. Appl. Phys.* **114**, 114907, (2013).

2 Selter, S., Bastien, G., Wolter, A. U. B., Aswartham, S. & Buchner, B. Magnetic anisotropy and low-field magnetic phase diagram of the quasi-two-dimensional ferromagnet $Cr_2Ge_2Te_6$. *Phys. Rev. B* **101**, 014440, (2020).

3 Khan, S. *et al.* Spin dynamics study in layered van der Waals single-crystal $Cr_2Ge_2Te_6$ *Phys. Rev. B* **100**, 134437, (2019).

4 Zeisner, J. *et al.* Magnetic anisotropy and spin-polarized two-dimensional electron gas in the van der Waals ferromagnet $Cr_2Ge_2Te_6$. *Phys. Rev. B* **99**, 165109, (2019).

5 MacNeill, D. *et al.* Control of spin–orbit torques through crystal symmetry in WTe_2/ferromagnet bilayers. *Nature Physics* **13**, 300-305, (2017).

6 Gong, C. *et al.* Discovery of intrinsic ferromagnetism in two-dimensional van der Waals crystals. *Nature* **546**, 265-269, (2017).

7 Lin, W. & Chien, C. L. Electrical Detection of Spin Backflow from an Antiferromagnetic Insulator/$Y_3Fe_5O_{12}$ Interface. *Phys. Rev. Lett.* **118**, 067202, (2017).

8 Shen, X. *et al.* Multi-domain ferromagnetic resonance in magnetic van der Waals crystals CrI_3 and $CrBr_3$. *J. Magn. Magn. Mater.* **528**, 167772, (2021).

9 Chen, Y. S., Lin, J. G., Huang, S. Y. & Chien, C. L. Incoherent spin pumping from YIG single crystals. *Phys. Rev. B* **99**, 220402, (2019).

10 Chen, L. *et al.* Emergence of anisotropic Gilbert damping in ultrathin Fe layers on GaAs(001). *Nat. Phys.* **14**, 490-494, (2018).

11 Li, Y. *et al.* Drag effect induced large anisotropic damping behavior in magnetic thin films with strong magnetic anisotropy. *J. Phys. Condens. Mater.* **33**, 175801, (2021).

12 Harder, M., Cao, Z. X., Gui, Y. S., Fan, X. L. & Hu, C. M. Analysis of the line shape of electrically detected ferromagnetic resonance. *Phys. Rev. B* **84**, 054423, (2011).

13 Zhou, H. *et al.* Spatial symmetry of spin pumping and inverse spin Hall effect in the Pt/$Y_3Fe_5O_{12}$ system. *Phys. Rev. B* **94**, 134421, (2016).

14 Arias, R. & Mills, D. L. Extrinsic contributions to the ferromagnetic resonance response of ultrathin films. *Phys. Rev. B* **60**, 7395-7409, (1999).

15 Zakeri, K. *et al.* Spin dynamics in ferromagnets: Gilbert damping and two-magnon scattering. *Phys. Rev. B* **76**, 104416, (2007).

16 Li, Y. *et al.* Giant Anisotropy of Gilbert Damping in Epitaxial CoFe Films. *Phys. Rev. Lett.* **122**, 117203, (2019).

17 Woltersdorf, G. & Heinrich, B. Two-magnon scattering in a self-assembled nanoscale network of misfit dislocations. *Phys. Rev. B* **69**, 184417, (2004).

18 Advanced Functional MaterialsConca, A., Keller, S., Schweizer, M. R., Papaioannou, E. T. & Hillebrands, B. Separation of the two-magnon scattering contribution to damping for the determination of the spin mixing conductance. *Phys. Rev. B* **98**, 214439, (2018).

19 Gupta, K., Wesselink, R. J. H., Liu, R., Yuan, Z. & Kelly, P. J. Disorder Dependence of Interface Spin Memory Loss. *Phys. Rev. Lett.* **124**, 087702, (2020).
20 Wen, Z., Kim, J., Sukegawa, H., Hayashi, M. & Mitani, S. Spin-orbit torque in Cr/CoFeAl/MgO and Ru/CoFeAl/MgO epitaxial magnetic heterostructures. *Aip. Adv.* **6**, 056307, (2016).
21 Shigematsu, E. *et al.* Significant reduction in spin pumping efficiency in a platinum/yttrium iron garnet bilayer at low temperature. *Appl. Phys. Exp.* **9**, 053002, (2016).
